# Thermoreversible [2 + 2] Photodimers of Monothiomaleimides and Intrinsically Recyclable Covalent Networks Thereof

**DOI:** 10.1021/jacs.4c04193

**Published:** 2024-07-02

**Authors:** Mohammed Aljuaid, Yujing Chang, David M. Haddleton, Paul Wilson, Hannes A. Houck

**Affiliations:** † Photochemistry for Materials Group, Department of Chemistry, University of Warwick, Library Road, Coventry CV4 7AL, United Kingdom; § Department of Chemistry, Turabah University College, Taif University, P.O. Box 11099, Taif 21944, Saudi Arabia

## Abstract

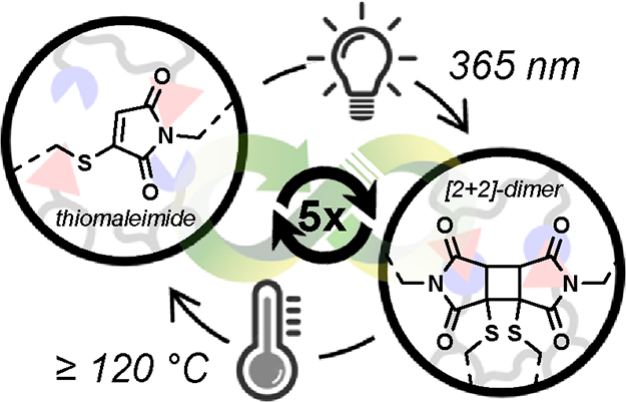

The development of intrinsically recyclable cross-linked materials remains challenged by the inherently unfavorable chemical equilibrium that dictates the efficiency of the reversible covalent bonding/debonding chemistry. Rather than having to (externally) manipulate the bonding equilibrium, we here introduce a new reversible chemistry platform based on monosubstituted thiomaleimides that can undergo complete and independent light-activated covalent bonding and on-demand thermal debonding above 120 °C. Specifically, repeated bonding/debonding of a small-molecule thiomaleimide [2 + 2] photodimer is demonstrated over five heat/light cycles with full conversion in both directions, thereby regenerating its initial monothiomaleimide constituents. This motivated the synthesis of multifunctional thiomaleimide reagents as precursors for the design of covalently cross-linked networks that display intrinsic switching between a monomeric and polymeric state. The resulting materials are shown to covalently dissociate and depolymerize upon heating both in solution and in bulk, thus transforming the densely photo-cross-linked material back into a viscous liquid. Temperature-regulated photorheology evidenced the intrinsic recyclability of the thiomaleimide-based thermosets during multiple cycles of UV cross-linking and thermal de-cross-linking. The thermally reversible photodimerization of thiomaleimides presents a new addition to the designer playground of dynamic polymer networks, providing interesting opportunities for the reprocessing and closed-loop recycling of covalently cross-linked materials.

## Introduction

Covalent network materials, such as thermosets, are indispensable for their superior durability and thermomechanical robustness but also cause ever-increasing concerns because of their chemically unrecyclable nature.^1^ Considerable research efforts have focused on improving the sustainability of polymer networks by replacing the permanent 3D cross-linked network structure with reversible bond connectivity.^2,3^ As a result, a plethora of covalent chemistry platforms have been developed to prepare dynamic polymer networks, thereby introducing material reshaping, healing, and reprocessing capabilities.^4−9^ Predominantly thermal activation of the embedded dynamic cross-links has been harvested to induce covalent bond rearrangement and reshuffling of the network structure. A fundamental limitation using thermal equilibrium reactions, however, is the inability to exclusively trigger either the bond forming or bond breaking process in an orthogonal manner.^10,11^ Hence, complete de-cross-linking and depolymerization back into the initial building blocks requires demanding reaction conditions (e.g., high temperatures) and/or additional chemicals (e.g., solvolysis) to shift the dynamic equilibrium toward the completely debonded state.^12,13^

An attractive strategy to externally and exclusively regulate and control either bond formation or dissociation is the use of photoreversible chemistries, as light brings high levels of directionality and selectivity. However, using light for both material cross-linking and de-cross-linking is often restricted to transparent thin films as the reverse photoreaction requires higher energy light, which limits the penetration depth.^14^ In addition, most photodynamic systems rely on homolytic chain cleavage (e.g., disulfides) that readily inflicts (irreversible) radical side reactions.^13,15^ Alternatively, light-gated dynamic covalent bonding/debonding has been achieved through photoreversible [2 + 2] and [4 + 4] dimerizations (A + A ⇌ A_2_), customarily making use of cinnamate, coumarin, and anthracene chemistry.^16^ Nonetheless, with recent advancements to shift the dimerization activation wavelength of such systems into the visible-light regime,^17−19^ degradation products are readily formed under the energetic UV conditions (i.e., λ < 300 nm) required to induce bond dissociation.^20,21^ Moreover, photodimer systems are dictated by a photostationary state and typically suffer from incomplete photocycloreversion caused by competitive light absorbance of the more conjugated scission products.^22^ The resulting incomplete covalent debonding can be compensated for at low concentrations,^23^ but this sets practical limitations to the reversible cross-linking and recycling of polymer networks such as bulk thermoset materials.

To address the challenges to design dynamic polymer networks with true closed-loop intrinsic recyclability, combining light-induced bond formations with thermally triggered bond reversions holds great potential. For instance, anthracene dimers are known to revert under UV irradiation but also under thermal conditions.^24,25^ In contrast to the photochemical bond scission, thermal reversion of the anthracene dimers does provide quantitative debonding; nonetheless, substantial heating (i.e., above 160 °C)^25^ also inflicts irreversible material damage and degradation.^21^ Orthogonal photochemical and “thermal” switching of covalent polymer networks has also been demonstrated using heteromolecular photocycloaddition chemistry.^26^ However, cross-linked materials are obtained only under continued visible-light irradiation as a result of the room-temperature instability of the formed cross-links. Developing thermoreversible photocycloadditions that display on-demand reversible bond formation under practically attainable and more relevant reaction conditions has yet to be achieved.

**Figure 1:**
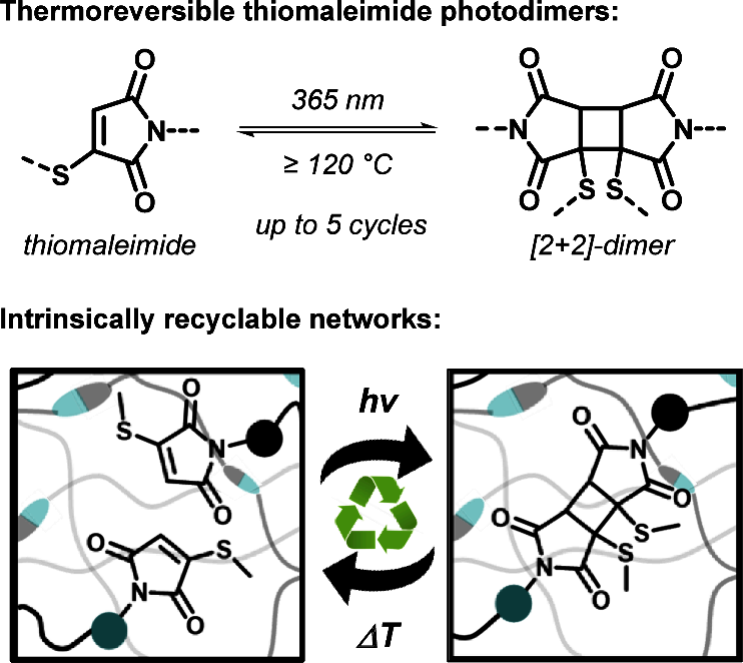
Introduction of thermoreversible thiomaleimide photodimers as a dynamic covalent chemistry, enabling the design of intrinsically recyclable networks.

Herein, we introduce a new dynamic covalent chemistry platform based on monosubstituted thiomaleimides that undergoes independent light-activated covalent bonding and on-demand-triggered debonding upon heating (Figure 1). In contrast to well-established conventional maleimides,^27,28^ the UV-induced [2 + 2] dimerization reaction of monosubstituted thiomaleimides has not been reported until 2012,^29^ since thiomaleimide photodimers have only been explored sporadically in peptide conjugation,^30^ polymer–polymer coupling,^31^ polyacrylamide hydrogel formation,^32^ and intramolecular cyclization.^33^ However, its potential to undergo reversible covalent bond (re)formation has not been reported. Here, we introduce for the first time that [2 + 2] photodimers of monothiomaleimides undergo a clean and quantitative thermal cycloreversion back into the initial starting reagents above 120 °C. The orthogonal bonding/debonding of mono- and multifunctional thiomaleimide compounds is investigated during consecutive cycles of UV irradiation and heating, both in solution and in bulk. The observed reversible switching between a liquid formulation and a covalently cross-linked network is further demonstrated, thereby highlighting the intrinsically recyclable nature of thiomaleimide-based thermosetting materials (Figure 1).

## Results and Discussion

**Figure 2:**
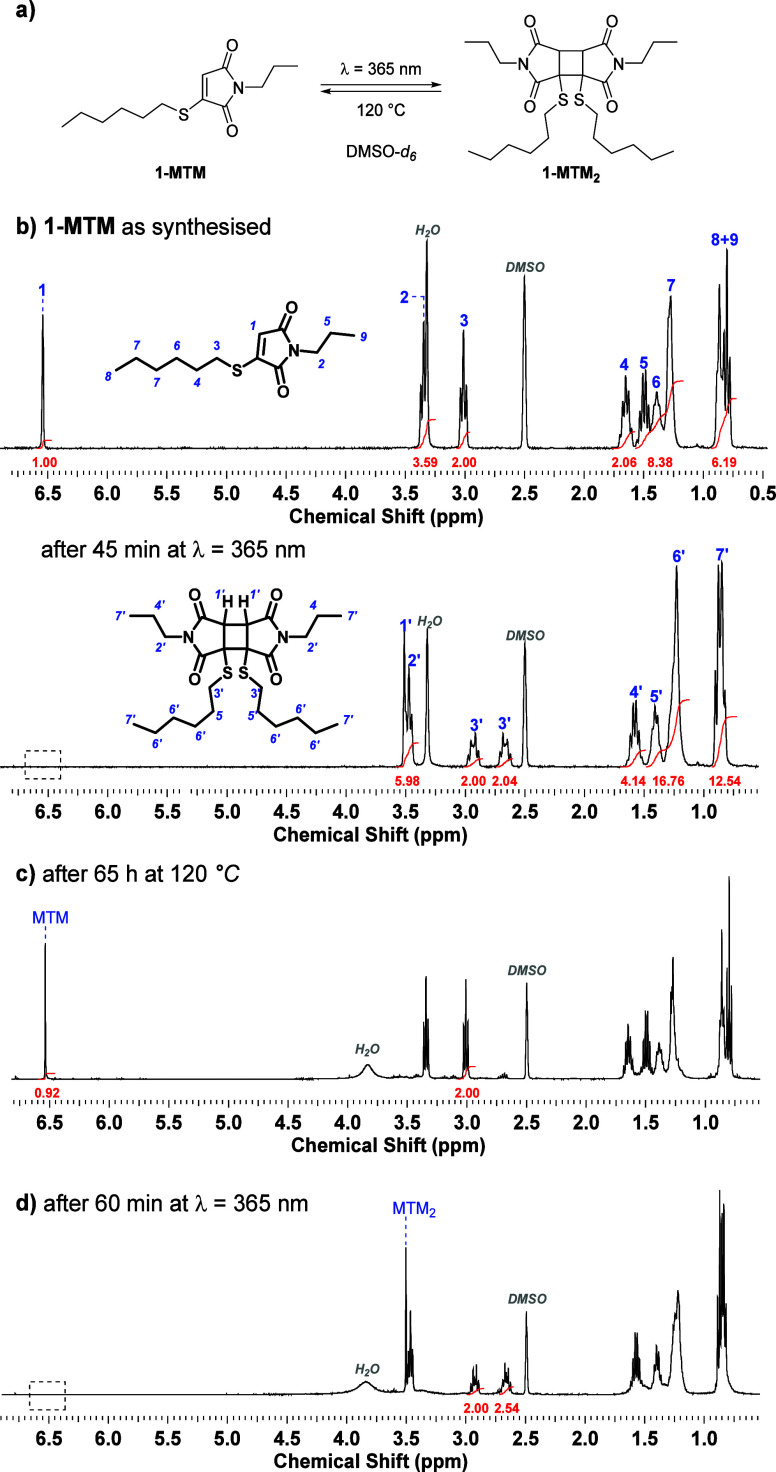
(a) Preliminary investigation of the thermal stability of thiomaleimide photodimer **1-MTM**_**2**_. (b) **1-MTM**_**2**_ was quantitatively preformed upon 45 min UV irradiation (λ = 365 nm) of synthesized **1-MTM** (10 mg in 0.5 mL of DMSO-*d*_*6*_), with characteristic ^1^H NMR resonances H1′ and H3′. (c) Heating **1-MTM**_**2**_ to 120 °C for 65 h regenerated the initial thiomaleimide **1-MTM**, (d) which was subsequently retransformed into its photodimer (λ = 365 nm, 60 min).

Our examination of the thermal stability of [2 + 2] thiomaleimide dimers was motivated by the understanding that in theory, every cycloaddition reaction is a reversible process, albeit often practically unattainable without also triggering competing degradation pathways. Thus, preliminary experiments were carried out initially to probe the stability of [2 + 2] thiomaleimide photodimers under both photochemical and thermal conditions. For this, a model compound dimer **1-MTM**_**2**_ (Figure 2a) was preformed by subjecting a solution of 3-(hexylthio)-*N*-propylmaleimide (**1-MTM**, 10 mg in 0.5 mL of DMSO-*d*_*6*_) to UV light-emitting diode (LED) irradiation for 45 min (λ = 365 nm, 15 mW cm^–2^, cf. Supporting Information). The formation of the photodimer was confirmed by ^1^H NMR spectroscopy, indicating the complete disappearance of the unsaturated C=C*H* proton resonance of the starting compound and the concomitant appearance of the characteristic cyclobutane and diastereotopic S–C*H*_2_ proton signals of the [2 + 2] cycloadduct (Figure 2b). The regioselective head-to-head conformation is corroborated with structural elucidation reported previously.^29^ Subsequent to its quantitative formation, the **1-MTM**_**2**_ photodimer was exposed to UV–C irradiation for 16 h (λ = 254 nm, 500 mJ cm^–2^, cf. Supporting Information), resulting in partial reformation of the starting thiomaleimide monomer, albeit in very low yield (i.e., 7%, derived from ^1^H NMR; see Figure S1). Importantly, however, considerable amounts of side products also formed during this prolonged exposure of the photodimer to high energy UV light, thus limiting its photoreversion potential.

In contrast to the partially observed photochemical cycloreversion, prolonged heating of the **1-MTM**_**2**_ solution at 120 °C (ca. 65 h, DMSO-*d*_*6*_) did result in the complete consumption of the thiomaleimide photodimer and a rather unexpectedly clean regeneration of the initial **1-MTM** monomer compound (cf. ^1^H NMR spectra, Figure 2c). To the best of our knowledge, this observed thermally induced retro-[2 + 2] reaction of monothiomaleimide photodimers has not been reported to date. Interestingly, [2 + 2] analogues of conventional maleimides did not display such a clean cycloreversion at elevated temperatures. Indeed, no trace of regenerated *N*-ethylmaleimide was observed upon heating the corresponding photodimer at 120 °C for 88 h (Figure S2). Moreover, ^1^H NMR analysis indicated thermal degradation and polymerization byproducts being formed upon heating the initial *N*-ethylmaleimide substrate (65 h at 120 °C in DMSO-*d*_*6*_, Figure S3). In contrast, no such thermal degradation or polymerization products were observed for the **1-MTM** model compound subjected to identical reaction conditions (Figure S4). Thermogravimetric analysis (TGA) and differential scanning calorimetry (DSC) further evidenced the differences in thermal stability of **1-MTM** compared to its unsubstituted *N*-ethyl maleimide counterpart (Figures S5 and S6). Subsequently, 1 h reirradiation at λ = 365 nm of the thermally regenerated **1-MTM** swiftly reformed the **1-MTM**_**2**_ dimer in quantitative yield (Figure 2d). Thus, thiol modification of the maleimide double bond enables an efficient and reversible covalent bonding behavior whereby bond (re)formation can be activated by UV light and debonding can be triggered upon heating.

Having verified the ability to regenerate thiomaleimides from their corresponding photodimers, the kinetics of both the forward photocycloaddition and thermal cycloreversion were investigated. Aliquots of a 50 mM stock solution of **1-MTM** in DMSO-*d*_*6*_ were distributed over several NMR tubes and irradiated for distinct periods of time with a UV LED array (λ = 365 nm, 0.5 W cm^–2^). Time-dependent conversions determined from the integration of the corresponding ^1^H NMR spectra indicated apparent first-order **1-MTM** photodimerization kinetics over the entire reaction course (Figure S7 and Table S1). This implies a reactant molecule in the excited state combining with one in the ground state with photoexcitation being the rate-limiting step,^34^ which aligns with the mechanism proposed by Malde et al.^33^ Full **1-MTM** conversion was achieved within 30 min, with an observed rate coefficient *k*_obs_ = 0.0675 min^–1^ and a reaction half-life time *t*_1/2_ of 10 min, underpinning the more efficient dimerization compared to its nonthiol-substituted derivative. Indeed, under identical conditions of irradiation, *N*-ethyl maleimide photodimerization required 3.5 h to reach 99% conversion (*t*_1/2_ = 32 min, Figure S8 and Table S2 for kinetics data). This difference in photoreactivity was suggested previously to result from a difference in absorption rather than a change in quantum yield.^33^

**Figure 3:**
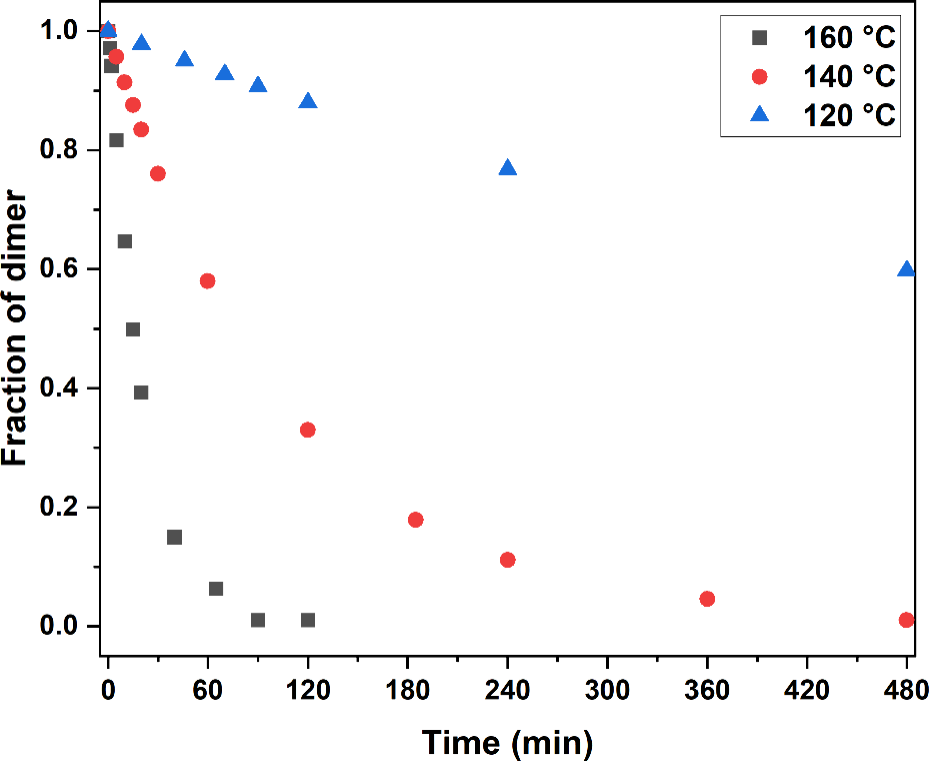
Cycloreversion kinetics of thiomaleimide photodimer **1-MTM**_**2**_ (0.025 M in DMSO-*d*_*6*_), derived from offline ^1^H NMR monitoring.

After kinetic profiling of the photo-[2 + 2]-cycloaddition, the reaction mixtures containing a quantitatively formed **1-MTM**_**2**_ dimer (25 mM in DMSO-*d*_*6*_) were placed in a preheated oil bath to monitor the cycloreversion over time via offline ^1^H NMR spectroscopy. The thermal reversibility profiles obtained at three distinct temperatures, i.e., 120, 140, and 160 °C, displayed first-order kinetics with observed half-life times *t*_1/2_ of 10.5 h, 1.3 h, and 14 min, respectively (Figure 3, Figure S9, and Tables S3–S5). An activation energy *E*_a_ = 133.8 ± 0.7 kJ mol^–1^ for the thermal cycloreversion of the thiomaleimide photodimers was extracted from the slope of the Arrhenius plot of the experimentally determined rate coefficients (i.e., ln *k* (*T*^–1^), Figure S10 and Table S6). This thermally attainable activation energy is believed to arise from the captodative stabilizing effect of the *R*–*S*-substituent on the 1,4-diradical intermediate that is formed during the nonconcerted cyclobutane scission (Figure S11).^35^ The derived activation energy for the formal thiomaleimide [2 + 2]-cycloreversion is comparable, yet lower, to the backward reaction barrier reported for the thermal dissociation of plain anthracene [4 + 4]-photodimers (ca. 155 kJ mol^–1^).^36^

The photoformation and thermal reversion of the thiomaleimide dimerization were investigated further to assess its potential for multiple covalent bonding, debonding, and rebonding. **1-MTM** (50 mM, DMSO-*d*_*6*_) was subjected to consecutive cycles of 30 min irradiation at λ = 365 nm (0.5 W cm^–2^) followed by 90 min heating at 160 °C. ^1^H NMR analysis before and after each step evidenced up to five cycles of successful photodimerization and thermal dissociation (Figures S12–S14). Despite a minor trace (i.e., <2% after five cycles) of an apparent oxidation byproduct having formed, the cycloaddition and cycloreversion remained unaffected and continued to proceed with full conversion of the thiomaleimide monomer and dimer, respectively.

**Figure 4:**
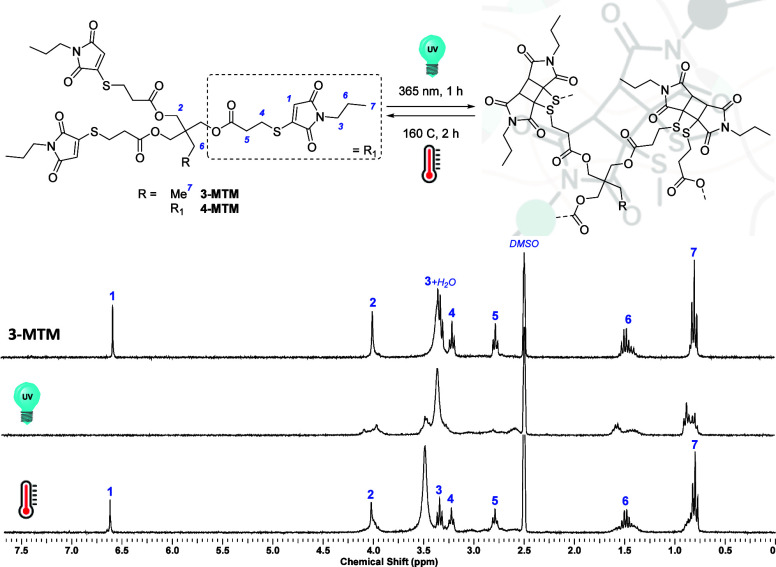
^1^H NMR investigation of the reversible cross-linking and de-cross-linking of a dilute solution of multifunctional thiomaleimide **3-MTM** (8 mg mL^–1^, DMSO-*d*_*6*_) under UV irradiation (λ = 365 nm, 1 h) and heating (160 °C, 2 h), respectively.

Once established, the newly introduced reversible bonding/debonding chemistry was explored for the design of intrinsically recyclable covalent polymer networks. Thus, the synthesis of multifunctional thiomaleimide-containing building blocks was targeted. Specifically, two novel tris- and tetra-functional thiomaleimide compounds, **3-MTM** and **4-MTM** (Figure 4), respectively, were prepared from *N*-propyl bromomaleimide—readily accessible from bromomaleic anhydride in a high-yielding one-step synthesis—and the corresponding commercial multivalent thiol precursor (see Figure S15 and the Supporting Information). An excess of the bromomaleimide reagent (i.e., 1.5 equiv per thiol) was used to suppress double thiol addition and ensure quantitative formation of the monothiol-substituted target compounds, as confirmed by ^1^H and ^13^C NMR spectroscopy and high-resolution mass spectrometry (Figures S16 and 17).

Following their multigram availability, test reactions were carried out to assess the photoreactivity of the multifunctional MTMs. Diluted solutions of **3-MTM** and **4-MTM** (8 mg mL^–1^, DMSO-*d*_*6*_) were subjected to offline ^1^H NMR analysis to evaluate structural changes before and after UV LED irradiation. Exposure of both multifunctional MTMs to UV light (1 h at λ = 365 nm, 15 mW cm^–2^) resulted in full conversion of the multifunctional reagents into their corresponding [2 + 2] photocycloaddition products with characteristic ^1^H NMR line broadening (Figure 4 and Figure S18). UV/vis spectra of **3-MTM** and **4-MTM** confirmed thiomaleimide consumption upon irradiation by the observed disappearance of the π → π* absorption band at λ_max_ = 354 nm (in DMSO, Figure S19). Heating the resulting **3-MTM** and **4-MTM** photoproduct solution for 2 h at 160 °C regenerated the thiomaleimide starting materials (Figure 4 and Figure S18, respectively). More concentrated solutions (0.2 g mL^–1^ in DMSO) displayed similar reversible transformations, as visually demonstrated (Figure S20) by the repeated gel formation of **3-MTM** after irradiation (λ = 365 nm, 2 h) and subsequent liquification after heating (160 °C, 1.5 h).

**Figure 5:**
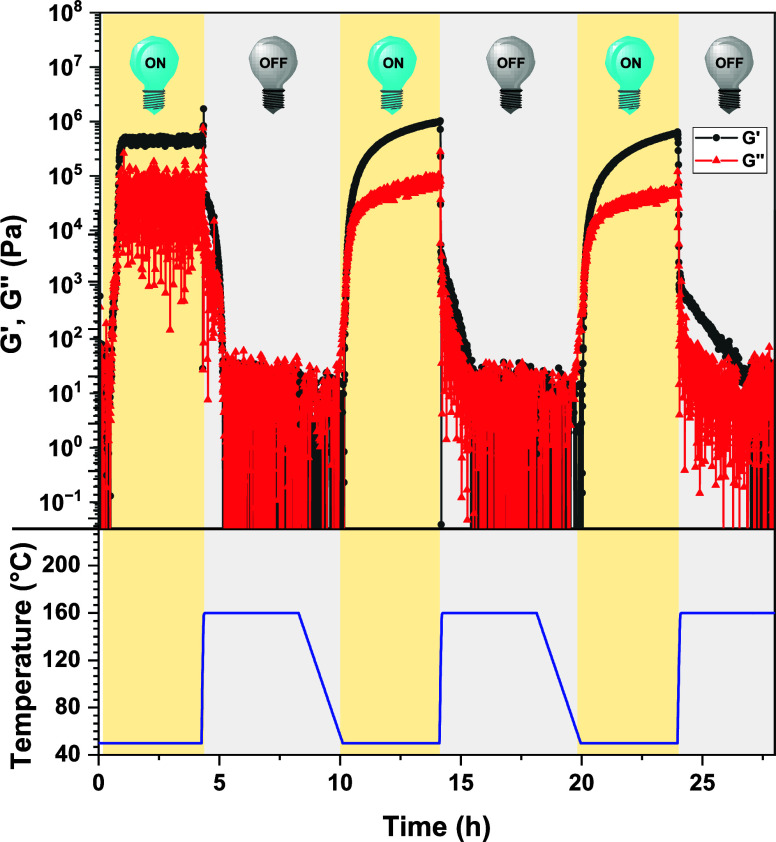
Thermal photorheology profile of **3-MTM** in bulk during consecutive cycles of UV irradiation (λ = 365 nm, 7 W cm^–2^, 4 h) and heating (160 °C, 4 h). Irradiation cycles were performed at slightly elevated temperature (i.e., 50 °C) to aid sample preparation of the viscous monomer.

Finally, the thermoreversible thiomaleimide photodimerization reaction was explored for the reversible synthesis of solvent-free covalent network materials. Thus, the viscoelastic response of both **3-MTM** and **4-MTM** was monitored via temperature-controlled photorheology measurements under oscillatory shear mode (cf. Supporting Information). Given the highly viscous appearance of the multifunctional thiomaleimide substrate, rheology measurements were initiated at a slightly elevated temperature, i.e., 50 °C, to ensure a homogeneous sample loading. The curing profile of **3-MTM**, depicted in Figure 5, displayed a low storage modulus *G*′ and loss modulus *G*″ during an initial period of 30 min (Figure S21). Irradiation with UV light (λ = 365 nm, 7 W cm^–2^), however, resulted in an abrupt increase of *G*′, thereby reaching a gel point within 10 min (Figure S21). A continued increase in storage modulus to 0.5 MPa was observed upon extended irradiation for 6 h, indicative of the formation of a photocured material (Figure 5). Subsequently, the resulting photo-cross-linked network was heated rapidly to 160 °C which triggered a sudden drop in *G*′ and *G*″. Both moduli continued to decrease and eventually returned to similar values of the pristine **3-MTM** starting material over the course of 2 h. Further heating totaling 12 h at 160 °C did not result in any change in the loss and storage modulus. A similar curing profile was observed for **4-MTM**, albeit reaching a slightly faster gel point after 8 min and a storage modulus plateau of 0.8 MPa (Figure S22). Thus, the thiomaleimide-based thermoset can be completely decross-linked by exploiting the intrinsic thermally reversible nature of the formed photocycloadduct.

In addition to the rheological investigation, further material characterization was carried out on dry thiomaleimide-based networks obtained after UV curing of **3-MTM** and **4-MTM** monomers. Infrared spectroscopy confirmed quantitative photoconversion of the multithiomaleimides and the regeneration of the initial monomers under bulk heating, as evidenced by the complete disappearance and the reappearance of the thio-substituted C=C stretch at 1556 cm^–2^ (Figures S23 and S24). Both networks showed degradation temperatures defined at 5% weight loss above 280 °C (Figure S25) and low swelling ratios (Table S7) in line with the expected densely cross-linked network structures derived from low molecular weight three- and four-arm monomers. Interestingly, the **3-MTM** and **4-MTM** monomers displayed a 50 °C upward shift in glass transition temperature upon photocuring (Figures S26 and S27), resulting in a network *T*_g_ of 47 and 68 °C, respectively, which is either slightly below or above the UV conditions applied at 50 °C during the photorheology experiments.

The UV-induced cross-linking and thermally triggered decross-linking processes of the thiomaleimide networks were repeated for two additional cycles to demonstrate the recyclability of the newly introduced materials. Reversible formation and dissociation of the **3-MTM-** and **4-MTM-**cross-linked networks were indeed evidenced by sequential transitions of a viscoelastic liquid into a cross-linked solid-like polymeric material during consecutive cycles of 4 h UV light and 4 h at 160 °C (Figure 5 and Figure S28, respectively). The covalently cross-linked networks were successfully reversed back into the initial formulation after each heating cycle. The four-arm-based network notably showed slower bulk decross-linking kinetics than its three-arm analogue, which can be rationalized by a higher average cross-linking density as indicated by the lower swelling ratio. Initially, a 12 h heating period was applied during the de-cross-linking step but was shown to result in a significant reduction of *G*′ during consecutive network reformation cycles (Figure S29). ^1^H NMR indicated some thermal thiomaleimide degradation during such extensive exposure to 160 °C (ca. 5%, Figure S30). Nonetheless, reducing the heating time to 4 h during each thermal step did result in similar storage moduli being obtained, thus indicating a recovery of cross-linking densities following UV reirradiation after each cycle (Figure 5 and Figure S28).

## Conclusions

In conclusion, our preliminary investigation led us to identify the thermally reversible nature of monosubstituted thiomaleimide photodimer compounds. The unprecedented thiomaleimide regeneration was studied by solution model reactions, indicating reversible transformations over several cycles of UV light and heating above 120 °C. The newly established covalent bonding, debonding, and rebonding chemistry platform was shown to enable the synthesis of intrinsically recyclable covalent networks both in diluted systems and under bulk thermosetting conditions. This was demonstrated with multifunctional thiomaleimide compounds that can be completely and repeatedly transformed from a viscous liquid into a cross-linked network and back, even under solvent-free conditions. Our findings introduce a new addition to the chemical toolbox to design covalent polymer networks that are not governed by a chemical equilibrium but can be orthogonally manipulated using light and temperature. In combination with its scaffold versatility, we posit that this will pave the way for further exploration of the thiomaleimide chemistry in sustainable material synthesis, including intrinsically recyclable thermosets.

## Supporting Information Available

Materials, instrumentation, supplementary figures and tables, and experimental and synthetic procedures (PDF)
