# Supplementary material for: Thermoreversible [2 + 2] Photodimers of Monothiomaleimides and Intrinsically Recyclable Covalent Networks Thereof
Source: J Am Chem Soc. 2024 Jul 2;146(28):19177–82. doi: 10.1021/jacs.4c04193 (PMC11258687; doi:10.1021/jacs.4c04193)
Supplement: Supplementary file 1 — ja4c04193_si_001.pdf [file ja4c04193_si_001.pdf]

Supporting Information File

Thermoreversible [2+2] photodimers of monothiomaleimides and intrinsically recyclable covalent networks thereof.

Mohammed Aljuaid,[†,§] Yujing Chang,[†] David M. Haddleton,[†] Paul Wilson,[†] and Hannes A. Houck,[†,*]

[†] Department of Chemistry, University of Warwick, Library Road, CV4 7AL Coventry, UK

[§] Department of Chemistry, Turabah University College, Taif University, P.O. Box 11099, Taif 21944, Saudi Arabia

[*] Hannes.Houck@warwick.ac.uk

Table of contents

Supplementary Figures and Tables 3
Supplementary Methods 25
Instrumentation 25
Materials 26
Experimental procedures 27
Preliminary photo- and thermal reversion studies of thiomaleimide photodimer **1-MTM$_2$** 27
N-Ethylmaleimide reference experiments 27
Photodimerization and cycloreversion kinetics 27
Determination of the cycloreversion rate coefficients and activation energy 28
Cyclability of MTM photodimerisation and thermal cycloreversion 29
Reversible crosslinking of thiomaleimide materials 29
Crosslinking/de-crosslinking in solution for ^{1}H-NMR analysis 29
Crosslinking/de-crosslinking in solution for gel formation 29
Crosslinking/de-crosslinking in bulk for rheology measurement 29
Synthetic procedures 30
Synthesis of thiomaleimide compounds 30
Synthesis of 3-(hexylthio)-1-propyl-1*H*-pyrrole-2,5-dione (3-(hexylthio)-*N*-propylmaleimide, **1-MTM**)[1] 30
Synthesis of **3-MTM** 30
Synthesis of **4-MTM** 31
Supplementary references 31

Supplementary Figures and Tables

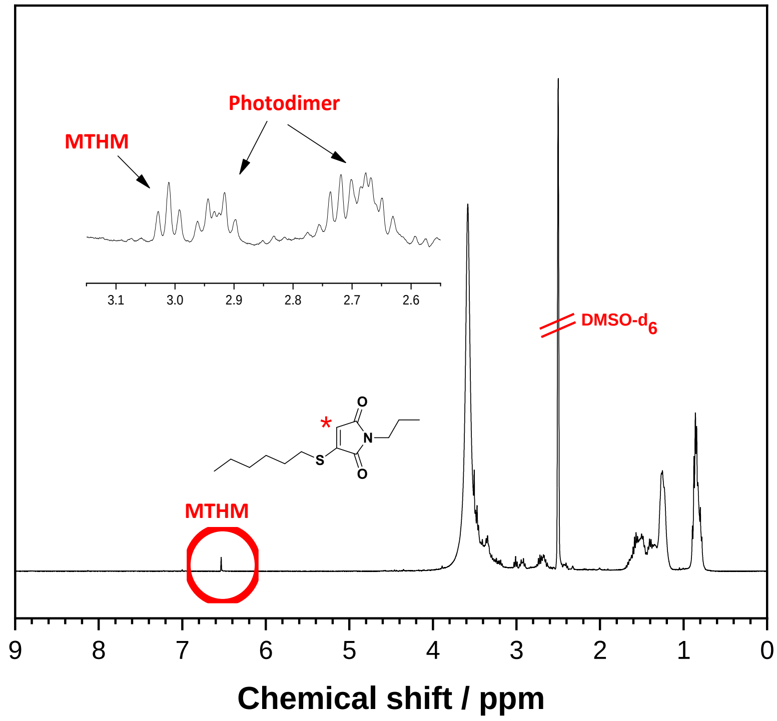

Figure S1. ^{1}H NMR spectrum (DMSO-*d*6) of 3-(hexylthio)-*N*-propylmaleimide photodimer **1-MTM2** after 16 hours of irradiated at λ_{max} = 254 nm.

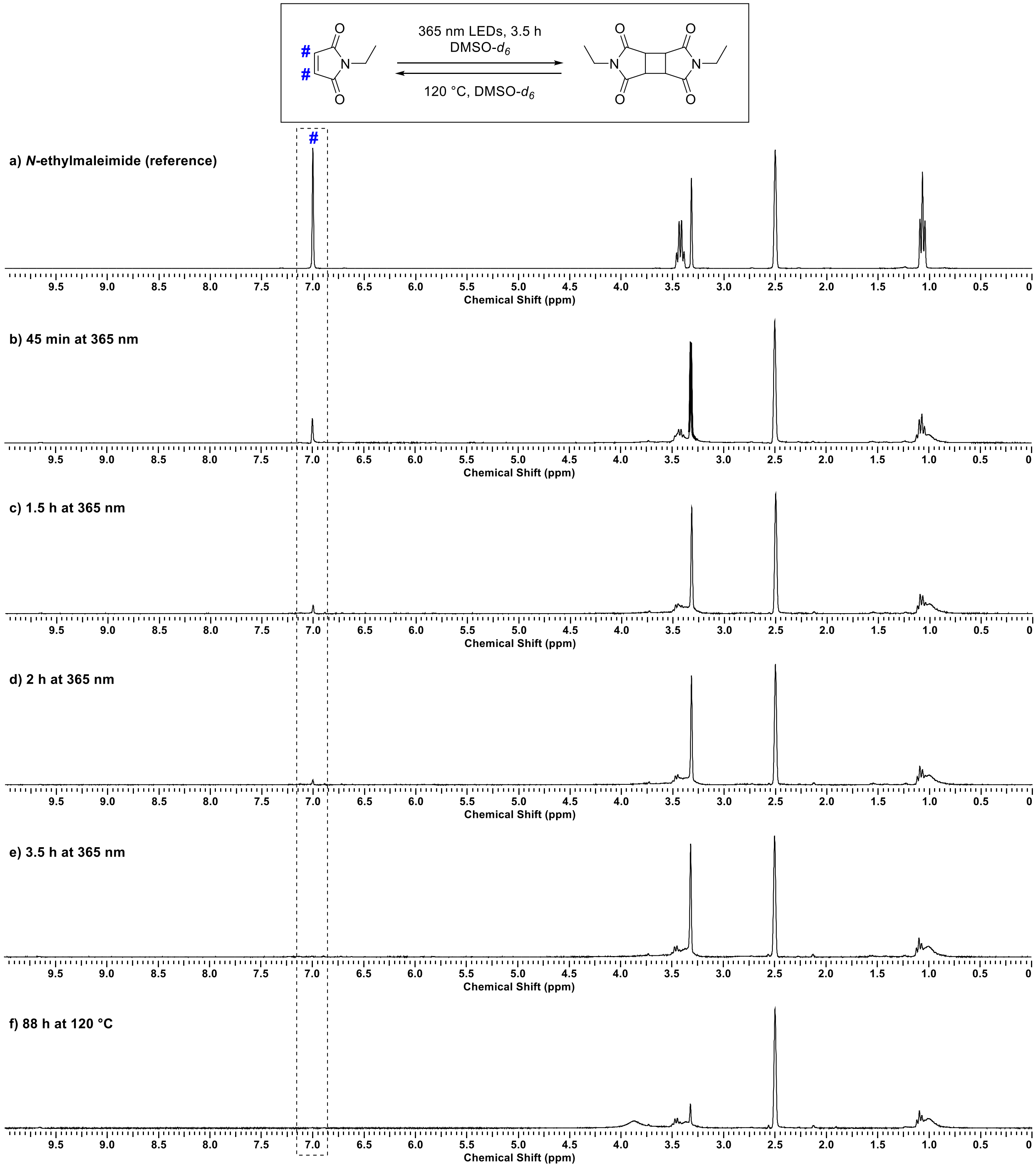

Figure S2. ^{1}H NMR spectra (DMSO-d_6) of (a) *N*-ethylmaleimide before, and (b-e) after photodimer formation under UV light (λ = 365 nm, 15 mW cm^{-2}, Lumidox LED array). (f) Extensive heating of the corresponding photodimer at 120 °C for 88 hours did not result in the reformation of the initial *N*-ethylmaleimide monomer.

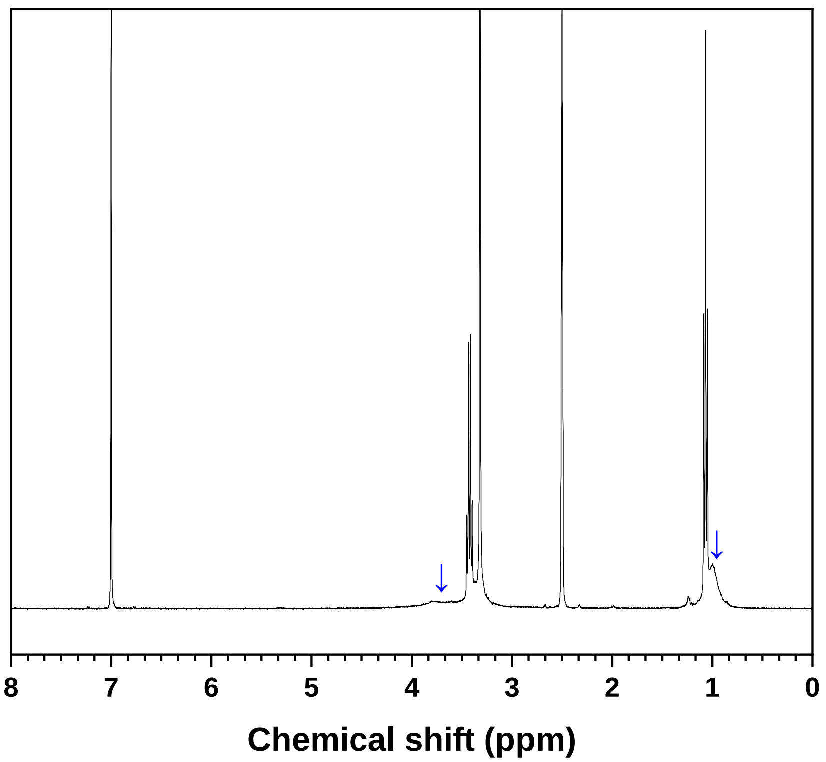

Figure S3. ^{1}H NMR spectrum (DMSO-d_6) of *N*-ethylmaleimide after heating at 120 °C for 65 hours.

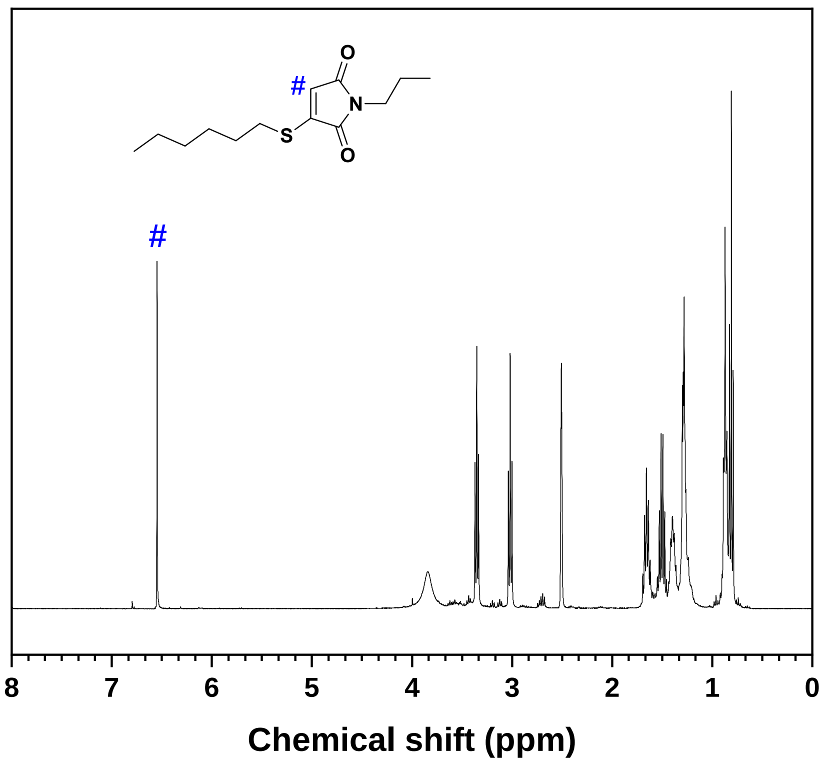

Figure S4. ^{1}H NMR spectrum (DMSO-d_6) of 3-(hexylthio)-*N*-propylmaleimide **1-MTM** after heating at 120 °C for 65 hrs.

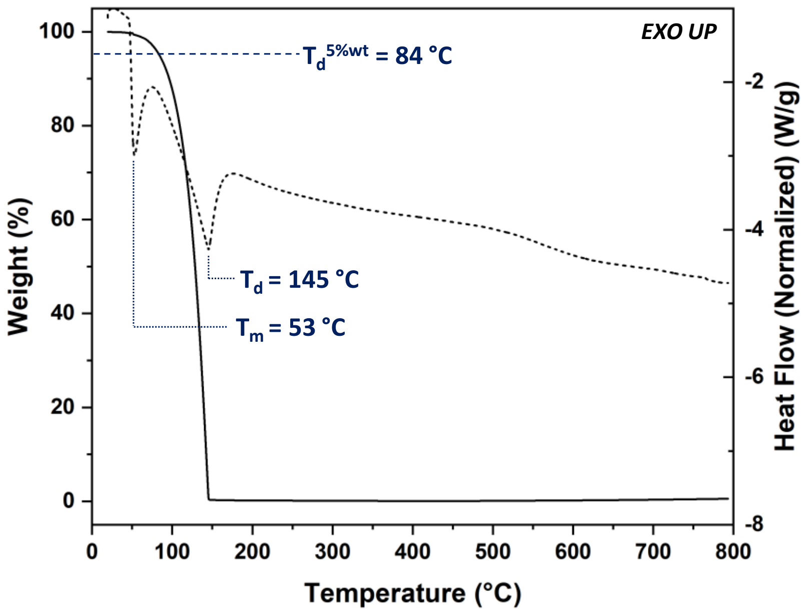

Figure S5. TGA (full line, left y-axis) and DSC thermogram (dashed line, right y-axis) of *N*-ethyl maleimide (**EtMal**), recorded under nitrogen atmosphere at a heating rate of 10 °C min^{-1}.

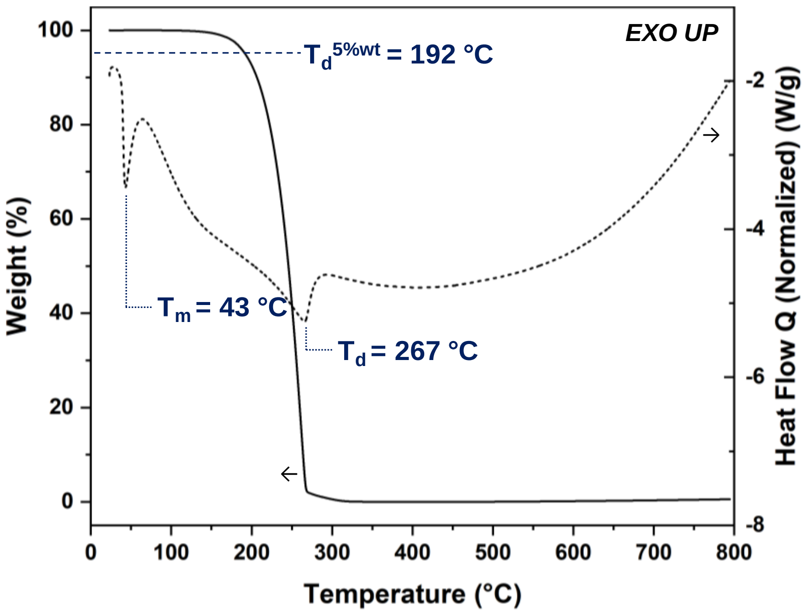

Figure S6. TGA (full line, left y-axis) and DSC thermogram (dashed line, right y-axis) of 3-(hexylthio)-*N*-propylmaleimide (**1-MTM**), recorded under nitrogen atmosphere at a heating rate of 10 °C min^{-1}.

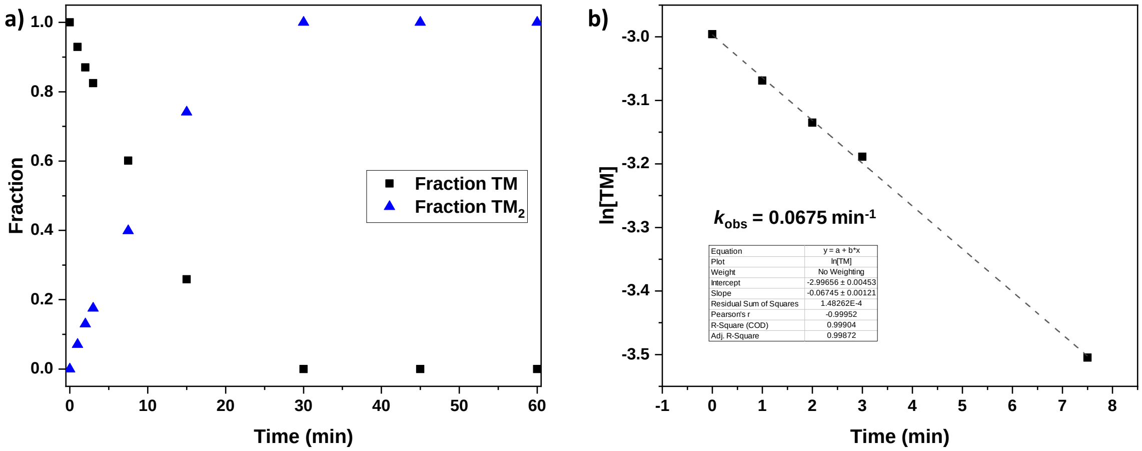

Figure S7. a) Cycloaddition kinetics of the [2+2] dimerization of 3-(hexylthio)-*N*-propylmaleimide (**1-MTM**, 50 mM in DMSO-d_6) upon irradiation with a UV LED array (λ = 365 nm, 0.5 W cm^{-2}). b) ln[**1-MTM**] as a function of time, indicating apparent 1st order reaction kinetics with an observed rate coefficient k_{obs} = 0.0675 min^{-1}.

Table S1. Fraction of remaining thiomaleimide **1-MTM** and formed [2+2] cycloadduct **1-MTM$_2$** upon irradiation of **1-MTM** (50 mM, DMSO-d_6) with a λ = 365 nm LED array (0.5 W cm^{-2}), as determined via ^{1}H NMR spectroscopy.

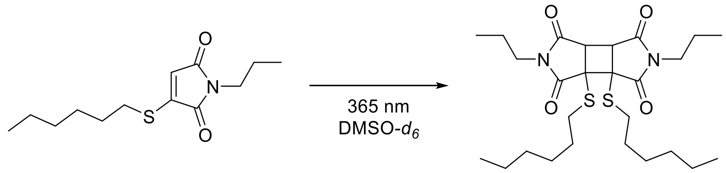

1-MTM **1-MTM$_2$**

Time (min)	Fraction **1-MTM**	Fraction **1-MTM$_2$**	Time (min)	Fraction **1-MTM**	Fraction **1-MTM$_2$**
0	1	0	15.0	0.259	0.742
1.0	0.930	0.071	30.0	< 0.001	> 0.999
2.0	0.870	0.130	45.0	< 0.001	> 0.999
3.0	0.825	0.176	60.0	< 0.001	> 0.999
7.5	0.601	0.399			

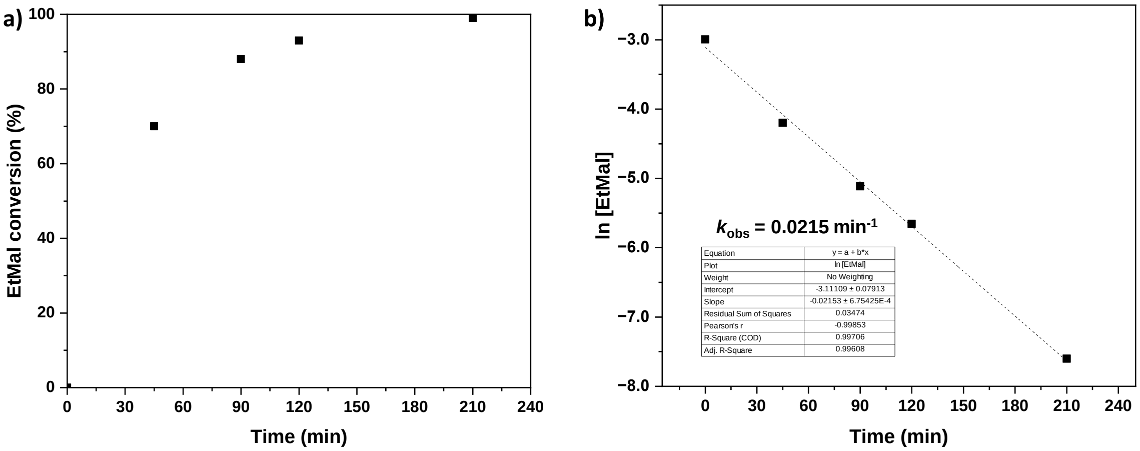

Figure S8. a) Cycloaddition kinetics of the [2+2] dimerization of *N*-ethylmaleimide (**EtMal**, 50 mM in DMSO-d_6) upon irradiation with a UV LED array (λ = 365 nm, 0.5 W cm^{-2}). b) ln[**EtMal**] as a function of time, indicating apparent 1st order reaction kinetics with an observed rate coefficient k_{obs} = 0.0215 min^{-1}.

Table S2. Fraction of remaining *N*-ethylmaleimide **EtMal** and formed [2+2] cycloadduct **EtMal₂** upon irradiation of **EtMal** (50 mM, DMSO-d_6) with a λ = 365 nm LED array (0.5 W cm^{-2}), as determined via ^{1}H NMR spectroscopy.

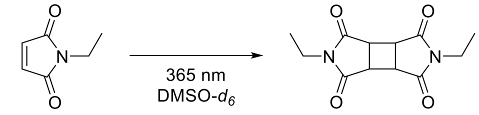

EtMal **EtMal$_2$**

Time (min)	Fraction **EtMal**	Fraction **EtMal$_2$**
0	1	0
45	0.30	0.70
90	0.12	0.88
120	0.07	0.93
210	0.01	0.99

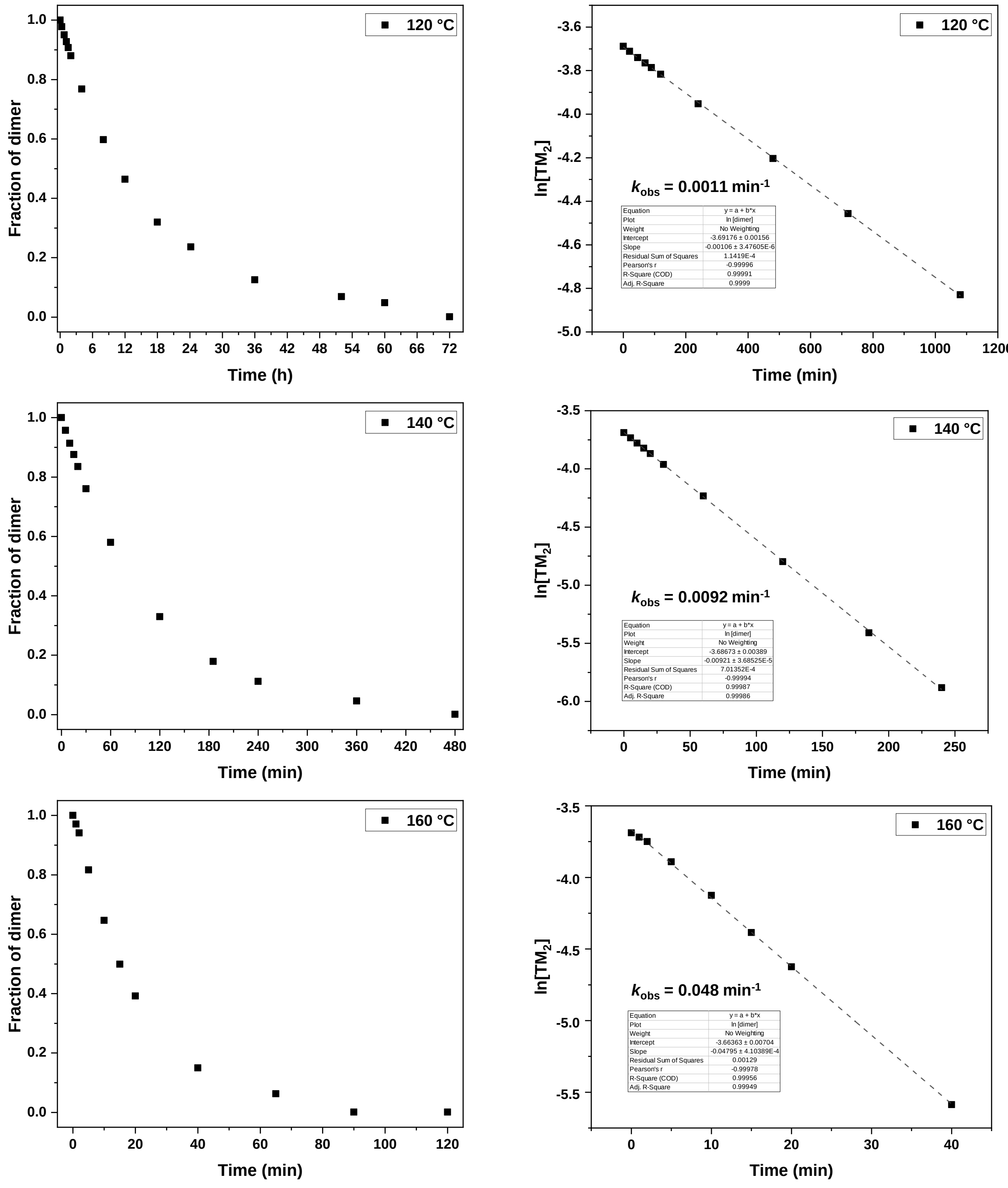

Figure S9. Thermal cycloreversion kinetics of the [2+2] dimer **1-MTM₂** (25 mM in DMSO-d_6) at 120 °C, 140 °C and 160 °C (left), and their corresponding 1st order kinetics profiles used to determine the observed rate coefficients k_{obs} (right).

Table S3. Fraction – determined via ^{1}H NMR spectroscopy – of remaining [2+2] cycloadduct **1-MTM**$_2$ and regenerated thiomaleimide **1-MTM** upon heating **1-MTM**$_2$ (25 mM, DMSO-d_6) at 120 °C. The photodimer **1-MTM**$_2$ was preformed by 45-min irradiation of **1-MTM** (50 mM, DMSO-d_6) with an LED array (λ = 365 nm, 0.5 W cm^{-2}).

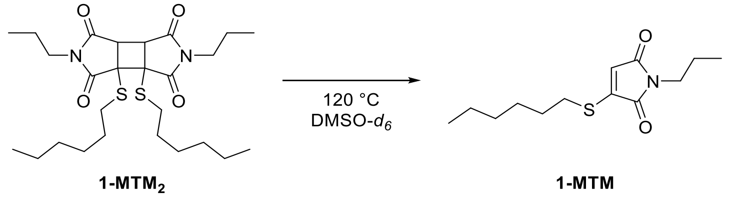

Time (h)	Fraction **1-MTM**$_2$	Fraction **1-MTM**	Time (h)	Fraction **1-MTM**$_2$	Fraction **1-MTM**
0	1	0	12.00	0.598	0.402
0.33	0.978	0.022	18.00	0.464	0.536
0.77	0.950	0.050	24.17	0.320	0.680
1.17	0.927	0.073	36.00	0.236	0.764
1.50	0.907	0.093	52.00	0.126	0.874
2.00	0.880	0.120	60.00	0.069	0.931
4.00	0.768	0.232	72.00	< 0.001	> 0.999
8.00	0.598	0.402			

Table S4. Fraction – determined via ^{1}H NMR spectroscopy – of remaining [2+2] cycloadduct **1-MTM**$_2$ and regenerated thiomaleimide **1-MTM** upon heating **1-MTM**$_2$ (25 mM, DMSO-d_6) at 140 °C. The photodimer **1-MTM**$_2$ was pre-formed by 45-min irradiation of **1-MTM** (50 mM, DMSO-d_6) with an LED array (λ = 365 nm, 0.5 W cm^{-2}).

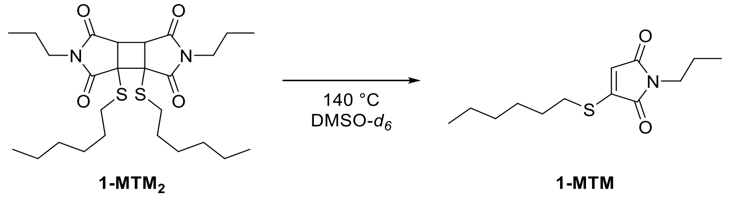

Time (min)	Fraction **1-MTM**$_2$	Fraction **1-MTM**	Time (min)	Fraction **1-MTM**$_2$	Fraction **1-MTM**
0	1	0	60	0.580	0.420
5	0.957	0.043	120	0.330	0.670
10	0.914	0.086	185	0.179	0.821
15	0.876	0.124	240	0.112	0.888
20	0.835	0.165	360	0.046	0.954
30	0.761	0.240	480	< 0.001	> 0.999

Table S5. Fraction – determined via ^{1}H NMR spectroscopy – of remaining [2+2] cycloadduct **1-MTM$_2$** and regenerated thiomaleimide **1-MTM** upon heating **1-MTM$_2$** (25 mM, DMSO-d_6) at 160 °C. The photodimer **1-MTM$_2$** was preformed by 45-min irradiation of **1-MTM** (50 mM, DMSO-d_6) with an LED array (λ = 365 nm, 0.5 W cm^{-2}).

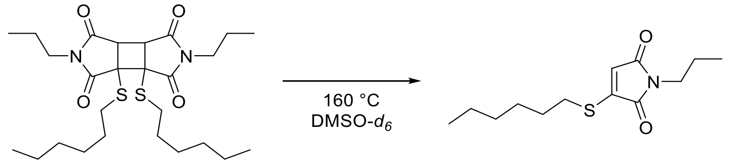

1-MTM$_2$ **1-MTM**

Time (min)	Fraction **1-MTM$_2$**	Fraction **1-MTM**	Time (min)	Fraction **1-MTM$_2$**	Fraction **1-MTM**
0	1	0	20	0.392	0.608
1	0.971	0.029	40	0.150	0.850
2	0.941	0.059	65	0.063	0.937
5	0.817	0.183	90	< 0.001	> 0.999
10	0.646	0.354	120	< 0.001	> 0.999
15	0.499	0.501			

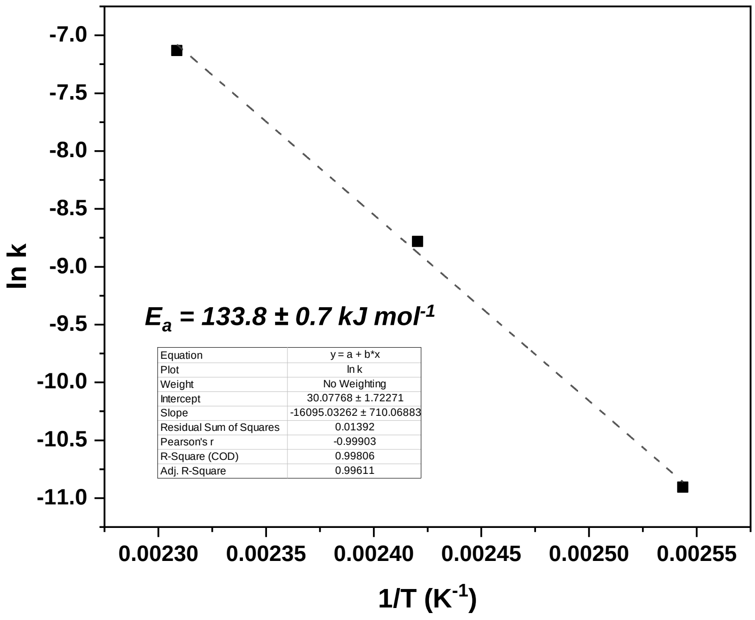

Figure S10. Arrhenius plot used to determine the activation energy for the thermal cycloreversion of **1-MTM**.

Table S6. Temperature-dependent rate coefficients and half-life times for the cycloreversion of thiomaleimide [2+2] cycloadduct **1-MTM$_2$**.

Temperature (°C)	Rate coefficient k (10^{-5} s^{-1})	Half-life time $t_{1/2}$ (min)	Half-life time $t_{1/2}$ (h)
120	1.83 ± 0.02	630.1	10.50
140	15.3 ± 0.06	75.3	1.26
160	80.0 ± 0.68	14.4	0.24

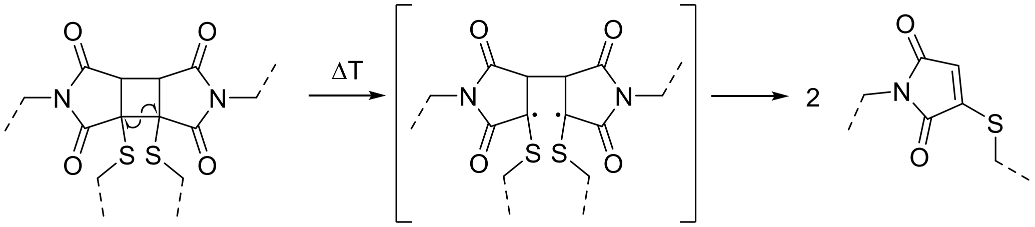

Figure S11. Rationalised stabilization of the 1,4-diradical intermediate during thermal reversion of thiomaleimide photodimers.

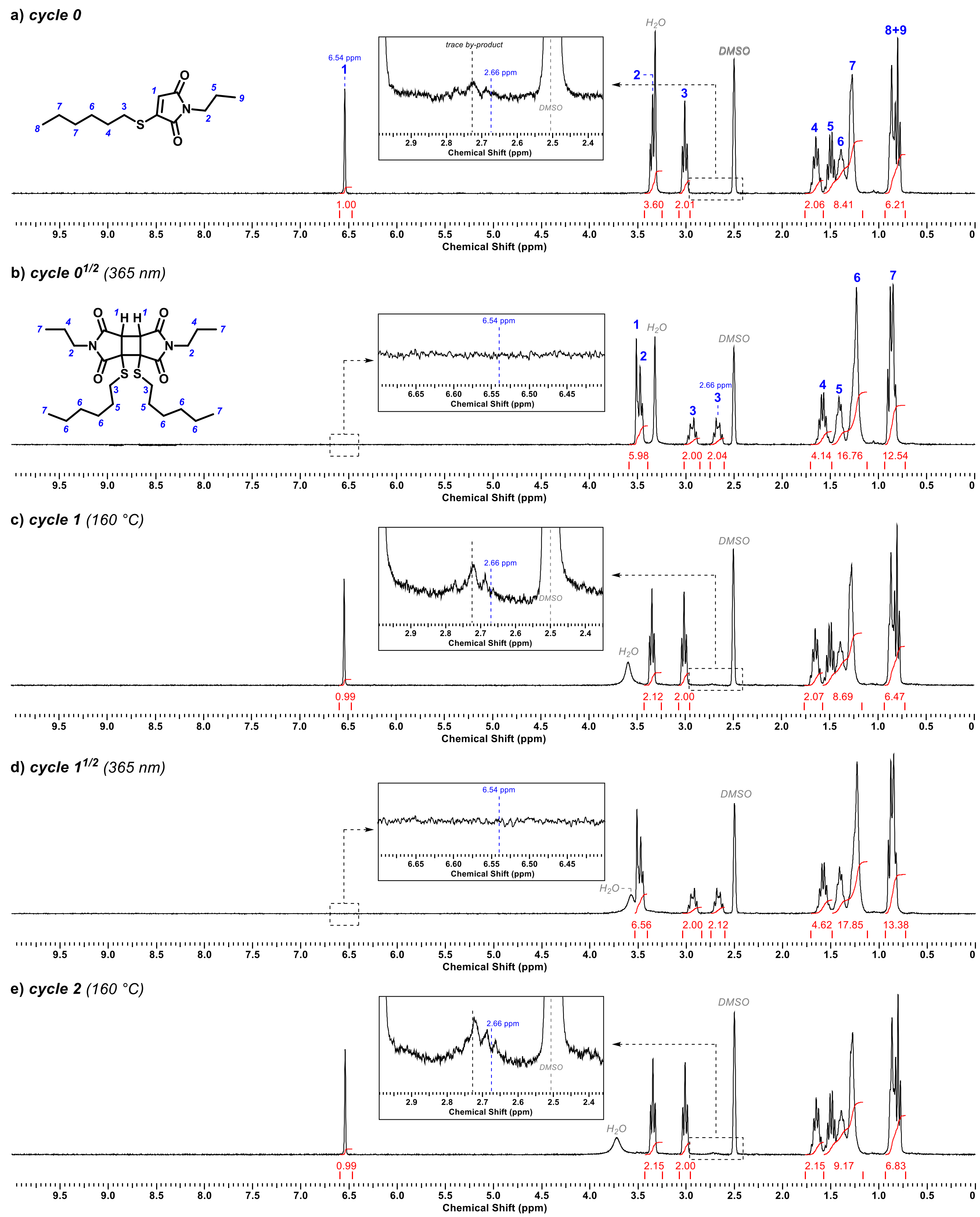

Figure S12. ^{1}H NMR spectra (DMSO-d_6) of the cyclic bonding/debonding/re-bonding experiment of thiomaleimide **1-MTM** (50 mM, DMSO-d_6) when subjected to consecutive cycles of 30 minutes irradiation at λ = 365 nm (0.5 W cm^{-2}, cycle $x^{1/2}$) and 90 minutes of heating at 160 °C (cycle x).

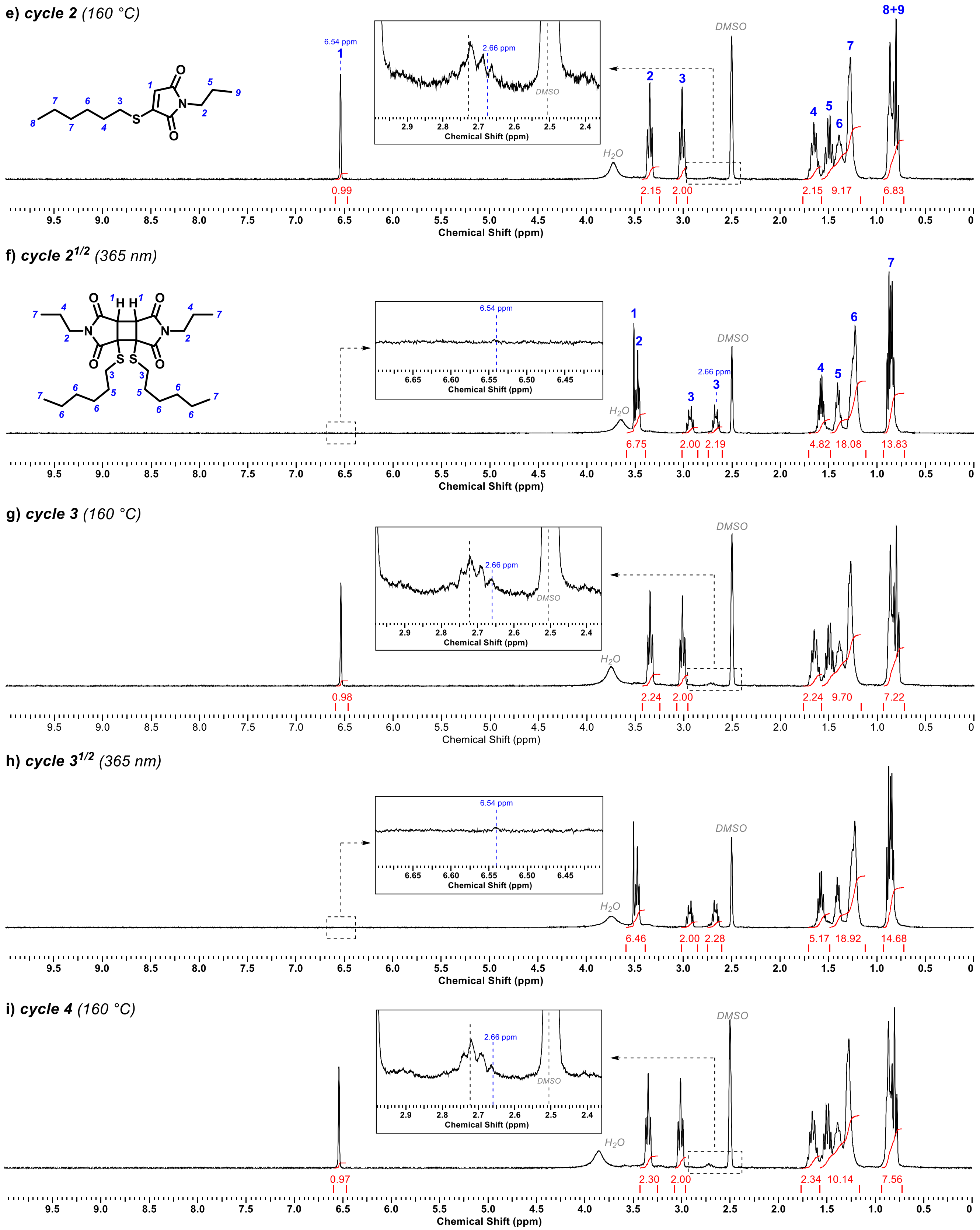

Figure S13. ^{1}H NMR spectra (DMSO-d_6) of the cyclic bonding/debonding/re-bonding experiment of thiomaleimide **1-MTM** (50 mM, DMSO-d_6) when subjected to consecutive cycles of 30 minutes irradiation at λ = 365 nm (0.5 W cm^{-2}, cycle $x^{1/2}$) and 90 minutes of heating at 160 °C (cycle x).

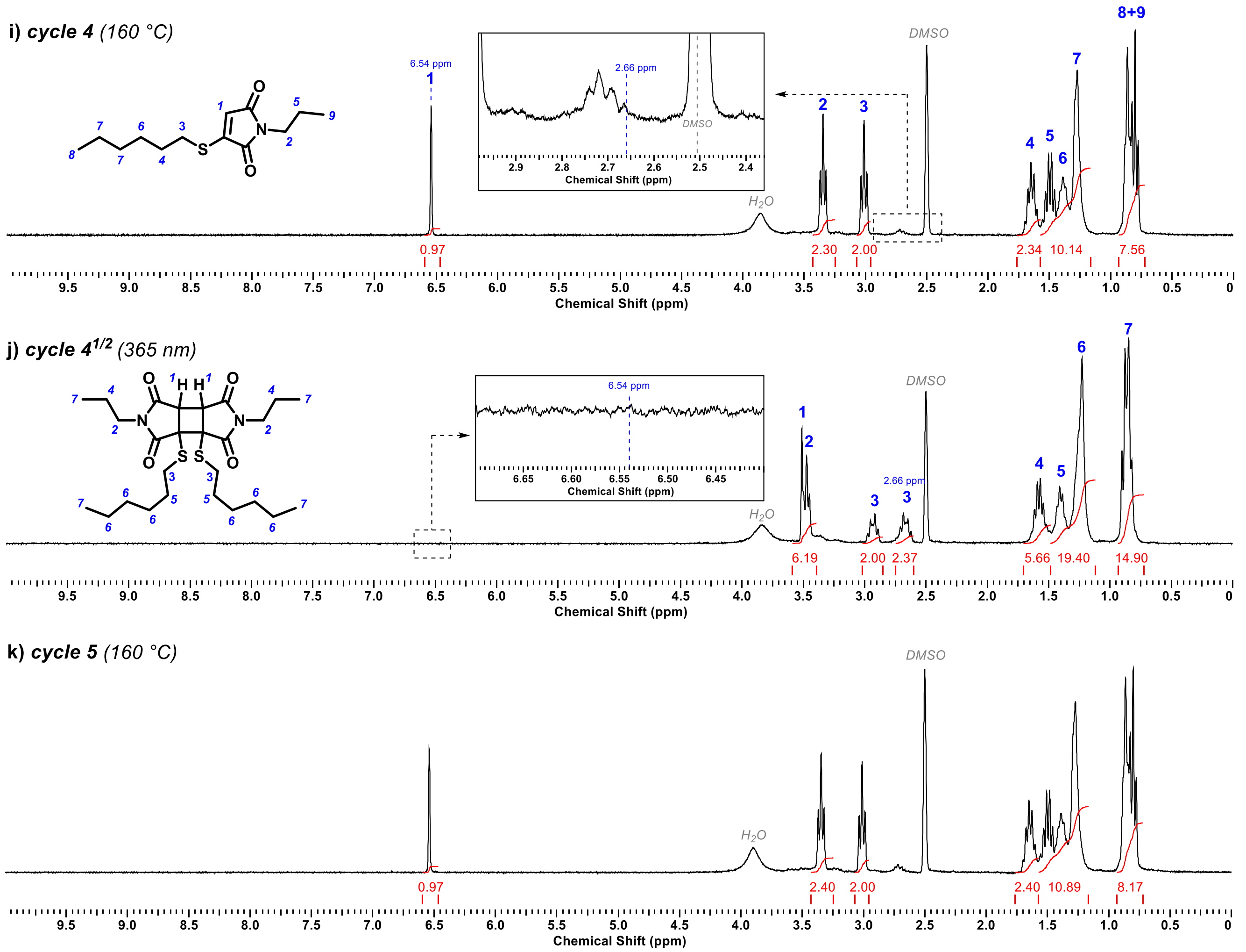

Figure S14. ^{1}H NMR spectra (DMSO-d_6) of the cyclic bonding/debonding/re-bonding experiment of thiomaleimide **1-MTM** (50 mM, DMSO-d$_6$) when subjected to consecutive cycles of 30 minutes irradiation at λ = 365 nm (0.5 W cm^{-2}, 'cycle $x^{1/2}$') and 90 minutes of heating at 160 °C ('cycle x').

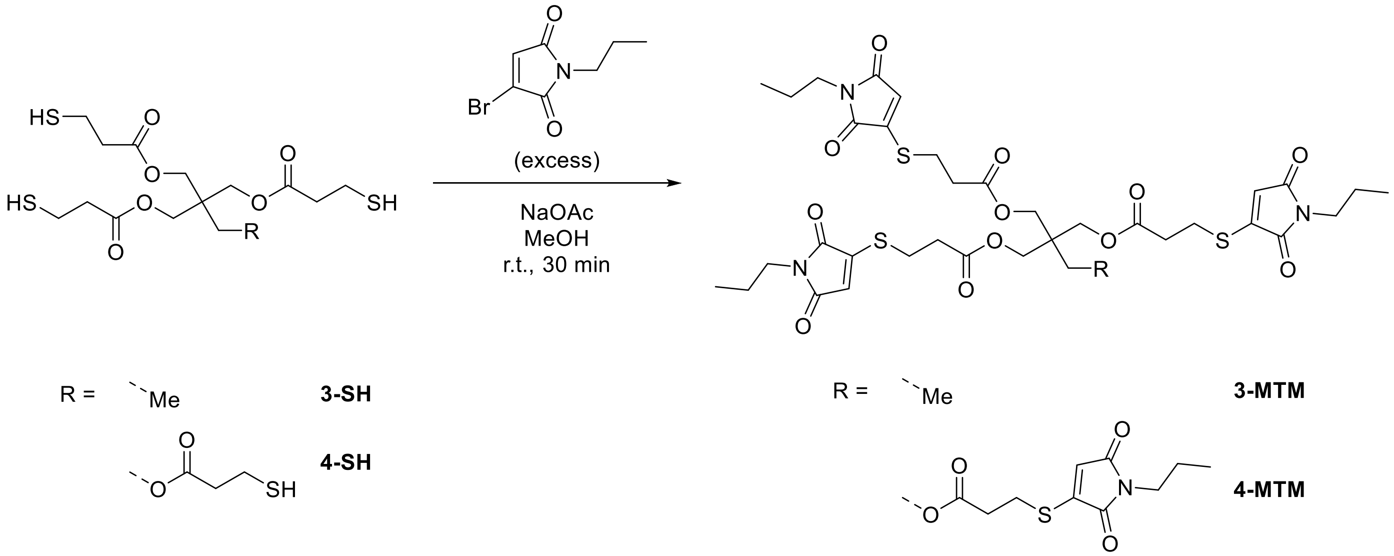

Figure S15. Synthesis of multi-functional thiomaleimide compounds **3-MTM** and **4-MTM** derived from commercially available thiol precursors.

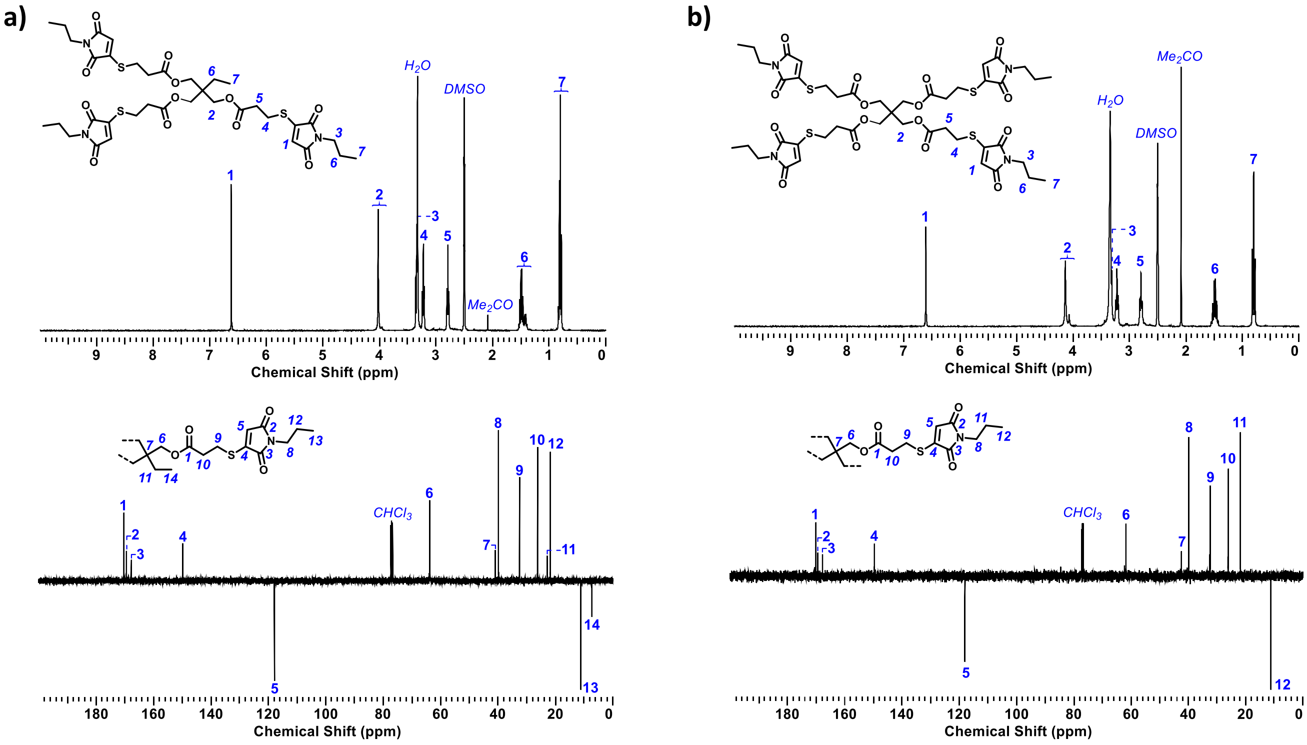

Figure S16. ^{1}H NMR (DMSO-d_6, top) and ^{13}C NMR ($CDCl_3$, bottom) spectra of synthesised (a) trisfunctional **3-MTM** and (b) tetrafunctional **4-MTM**.

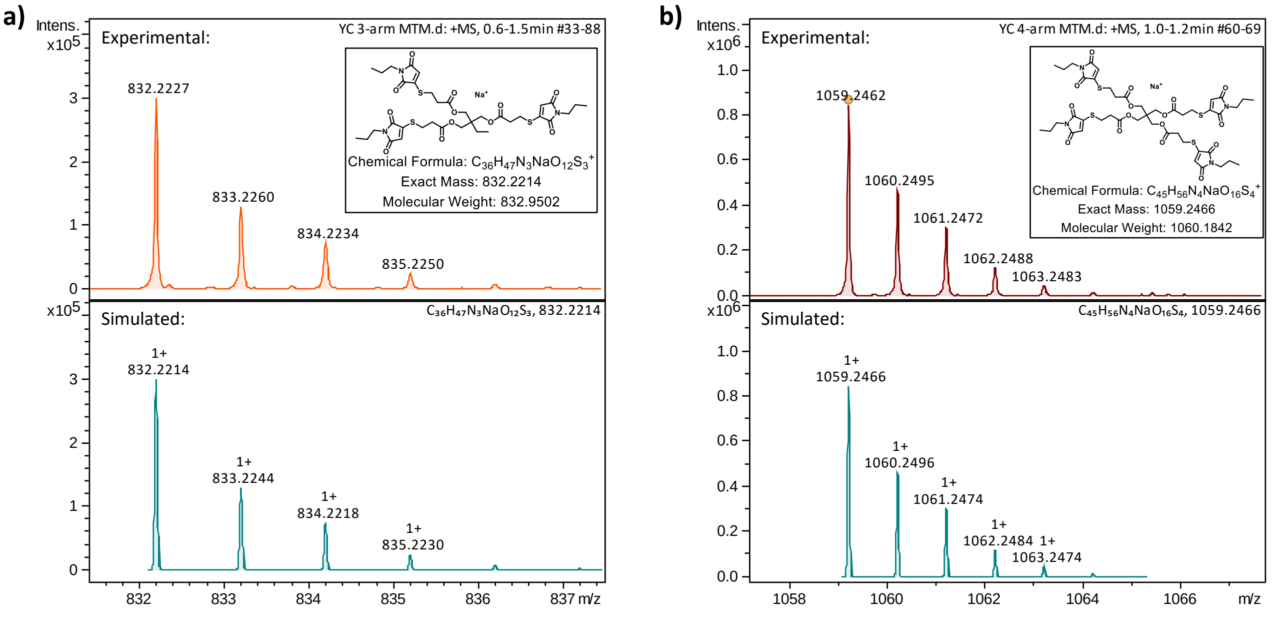

Figure S17. HR-ESI-MS of (a) synthesised trisfunctional **3-MTM** and (b) tetrafunctional thiomaleimide **4-MTM** (top: as found, bottom: calculated).

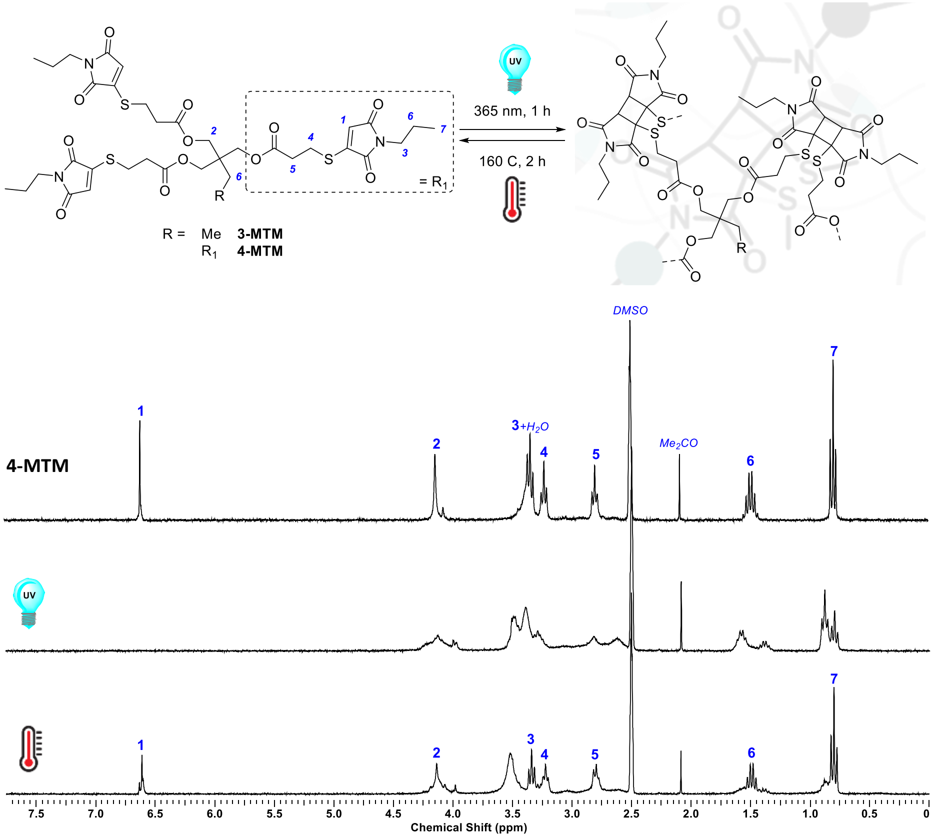

Figure S18. ^{1}H NMR investigation of the reversible crosslinking and decrosslinking of a dilute solution of multifunctional thiomaleimide **4-MTM** (8 mg mL-1, DMSO-d6) under UV irradiation (λ = 365 nm, 1 h) and heating (160 °C, 2 h), respectively.

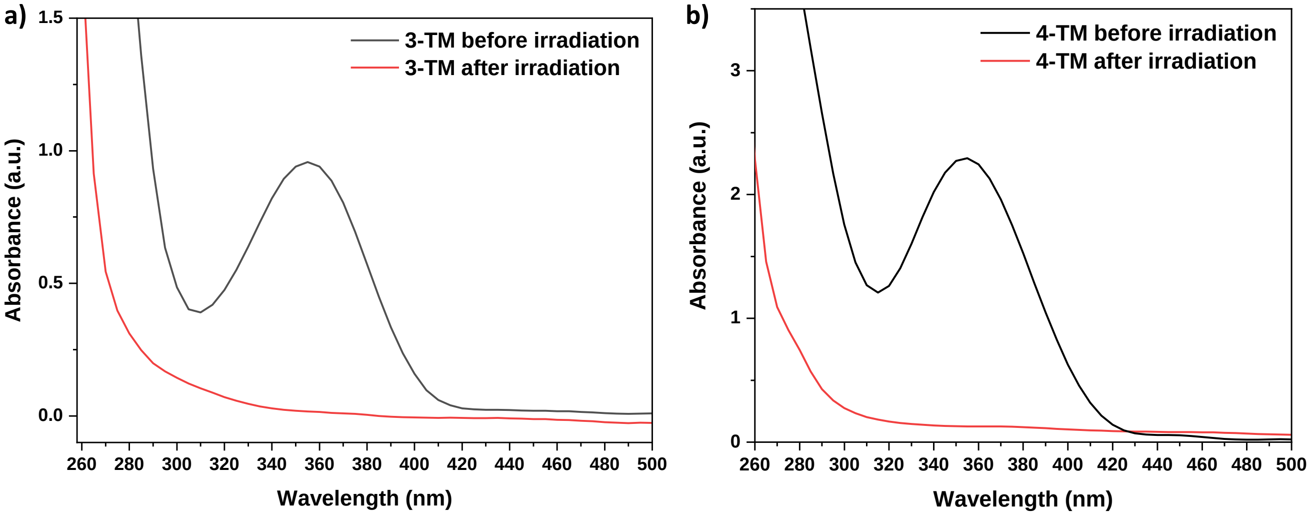

Figure S19. UV/vis spectra (DMSO) of (a) **3-MTM** and (b) **4-MTM** before and after UV irradiation (λ = 365 nm).

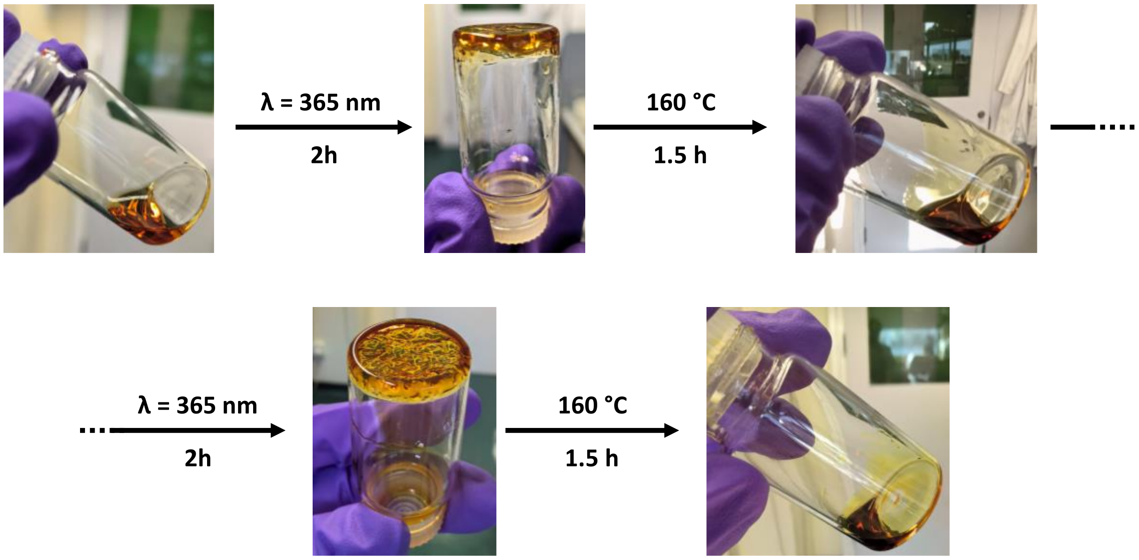

Figure S20. Reversible gelation of **3-MTM** (0.2 g mL^{-1} in DMSO) during two consecutive cycles of UV irradiation (2 h, λ = 365 nm, 30 mW cm^{-2}) and heating (1.5 h, 160 °C).

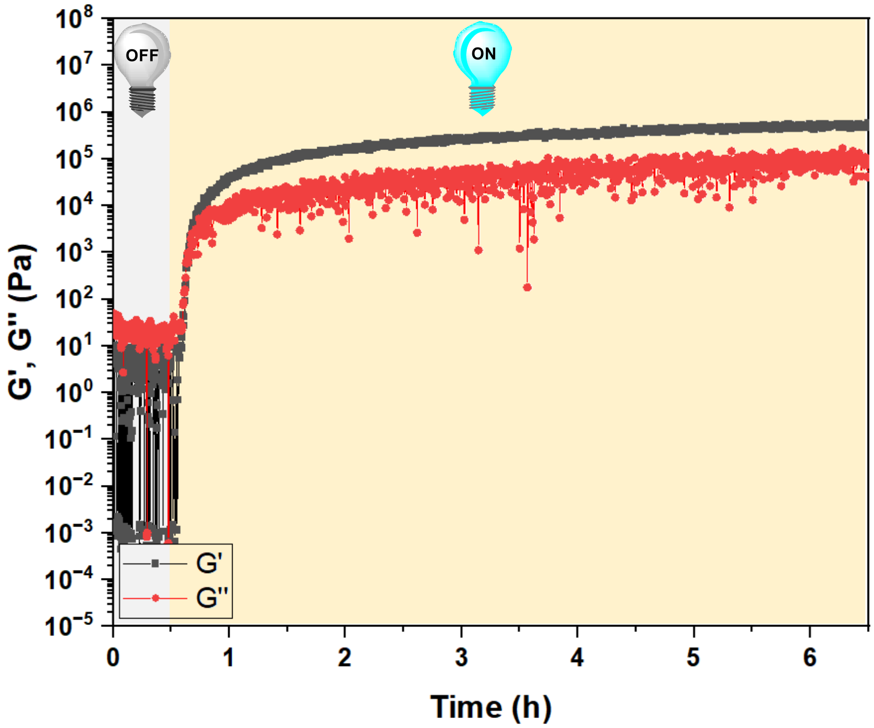

Figure S21. Thermal photorheology profile of **3-MTM** in bulk at 50 °C during an initial 30-minute period in the dark and the first cycle of 6 hours UV irradiation (λ = 320 – 390 nm), with a crossover of storage and loss modulus obtained within 10 minutes of irradiation.

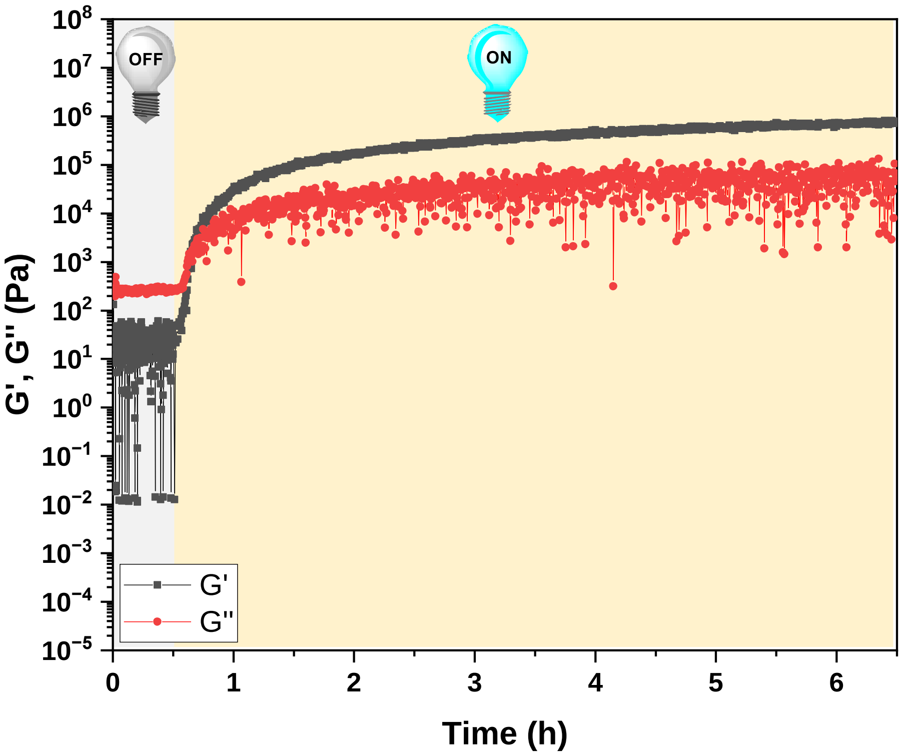

Figure S22. Thermal photorheology profile of **4-MTM** in bulk at 50 °C during an initial 30-minute period in the dark and the first cycle of 6 hours UV irradiation (λ = 320 – 390 nm), with a crossover of storage and loss modulus obtained within 8 minutes of irradiation.

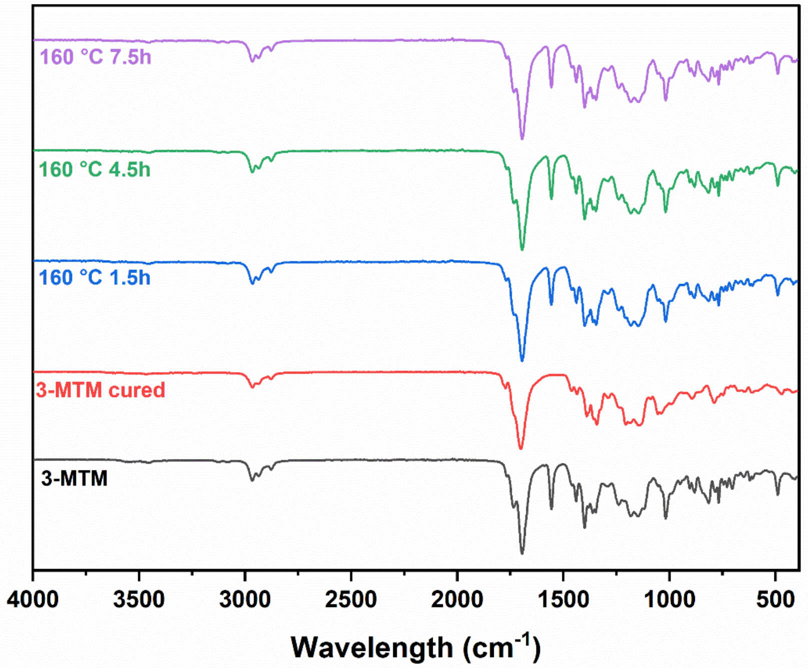

Figure S23. Infrared spectra of **3-MTM** before and after bulk UV-curing (λ = 365 nm), and during bulk decrosslinking at 160 °C.

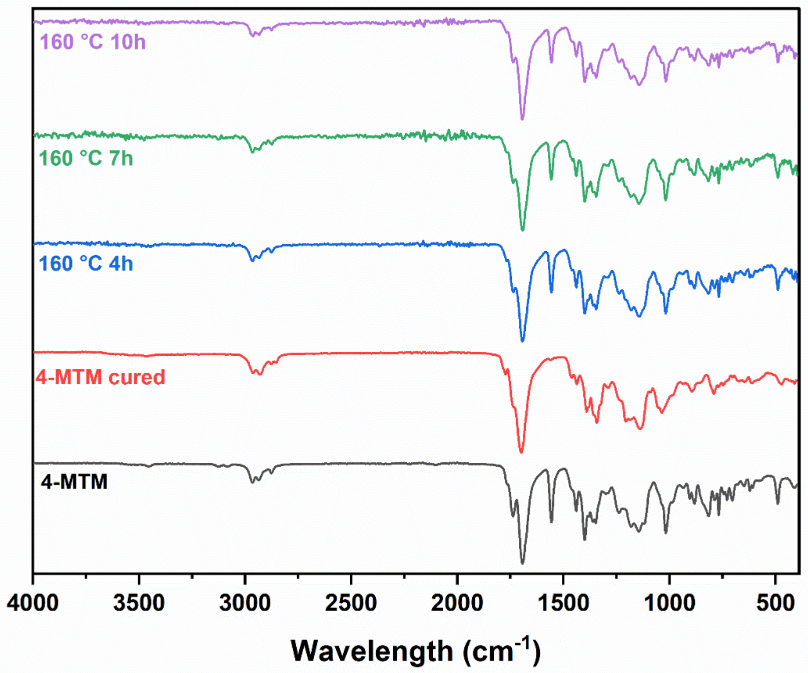

Figure S24. Infrared spectra of **4-MTM** before and after bulk UV-curing (λ = 365 nm), and during bulk decrosslinking at 160 °C.

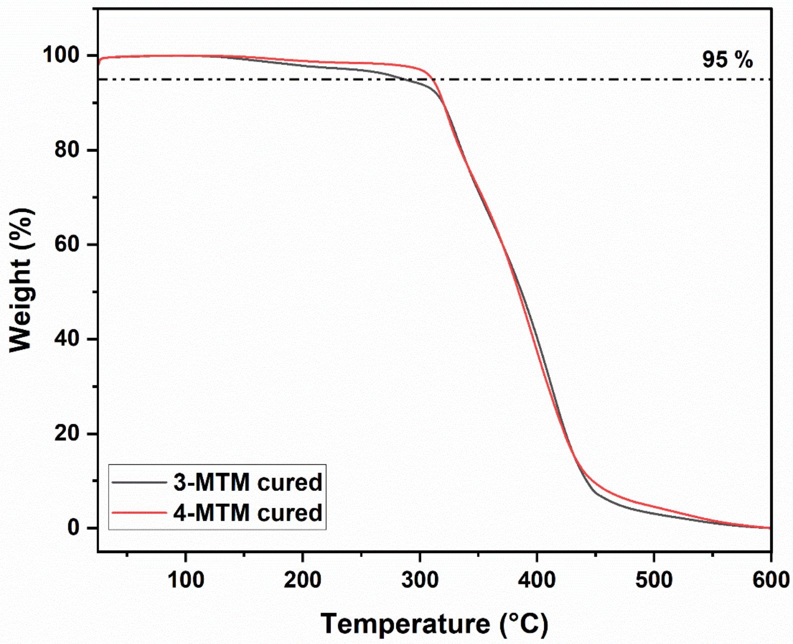

Figure S25. Thermogravimetric analysis of **3-MTM** and **4-MTM** networks obtained after bulk photo-curing at λ = 365 nm, recorded under nitrogen atmosphere at a heating rate of 10 °C min^{-1}..

Table S7. Swelling ratios of multi-functional thiomaleimide networks in different solvent after 7 days at room temperature, determined by gravimetry of the dried and swollen gels.

Solvent	Swelling ratio **3-MTM** network (%)	Swelling ratio **4-MTM** network (%)
methanol	0.8	0
dimethyl sulfoxide	10.0	13.5
acetone	4.3	3.5
water	18.0	2.9
dichloromethane	1.0	0

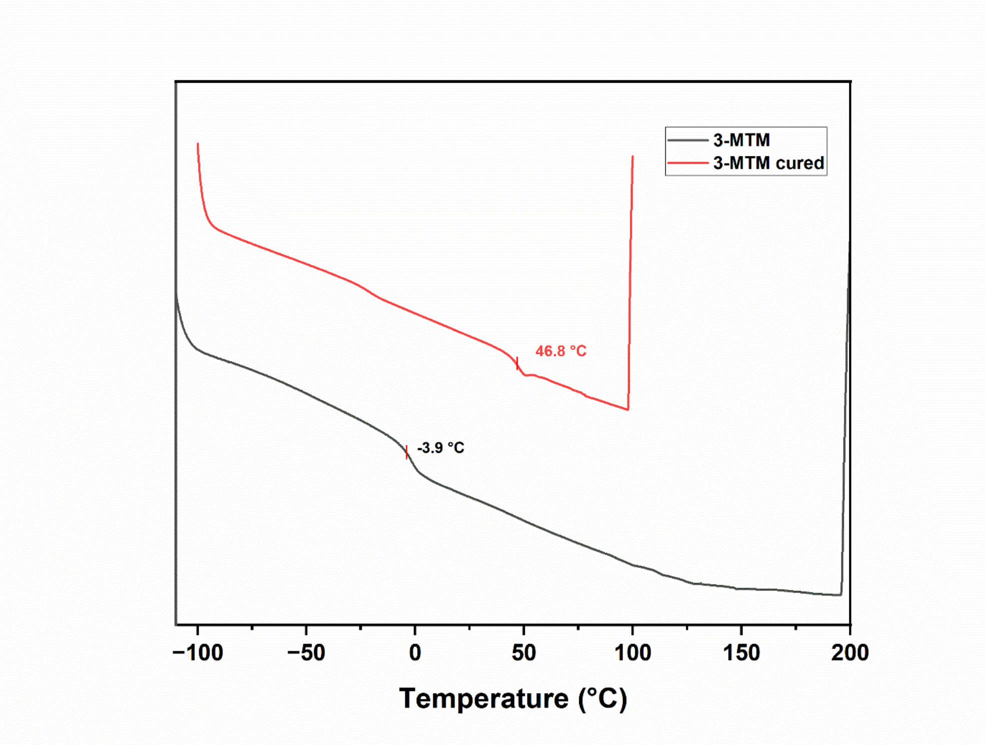

Figure S26. DSC trace (2nd heating, endo down) of **3-MTM** monomer (black, bottom) and UV-crosslinked **3-MTM** network (red, top), recorded under nitrogen atmosphere at a heating rate of 5 °C min^{-1}.

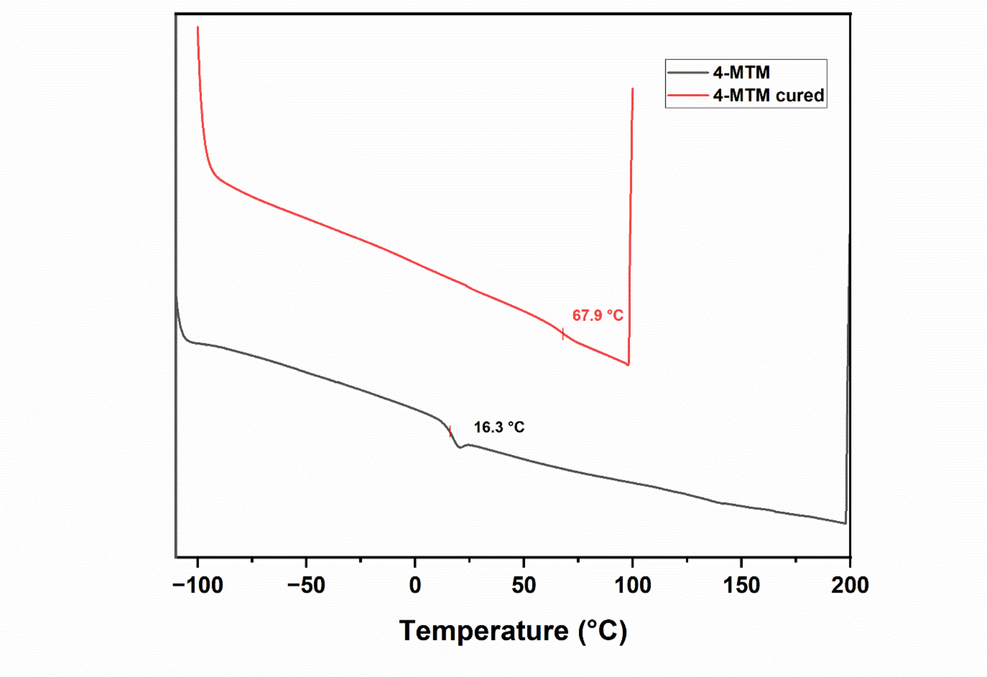

Figure S27. DSC trace (2nd heating, endo down) of **4-MTM** monomer (black, bottom) and UV-crosslinked **4-MTM** network (red, top), recorded under nitrogen atmosphere at a heating rate of 5 °C min^{-1}.

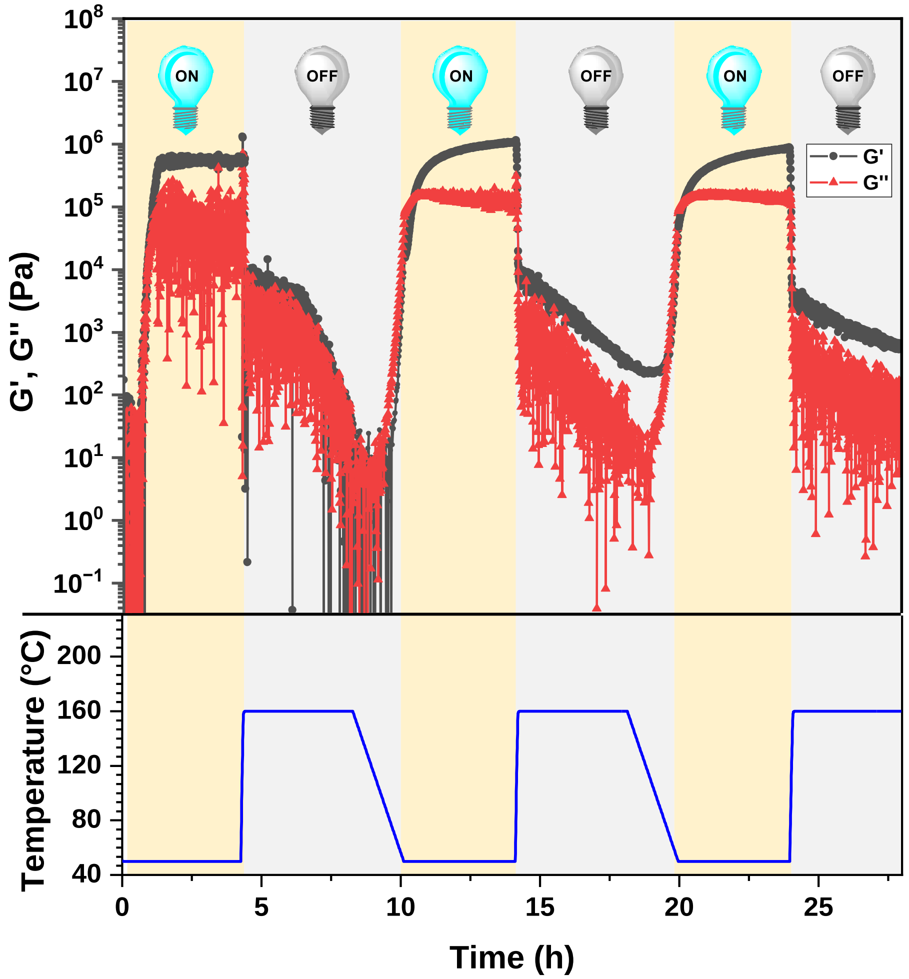

Figure S28. Thermal photorheology profile of **4-MTM** in bulk during consecutive cycles of UV irradiation (λ = 365 nm, 7 W cm^{-2}, 4 h) and heating (160 °C, 4 h). Irradiation cycles were performed at slightly elevated temperature (i.e. 50 °C) to aid sample preparation of the viscous monomer.

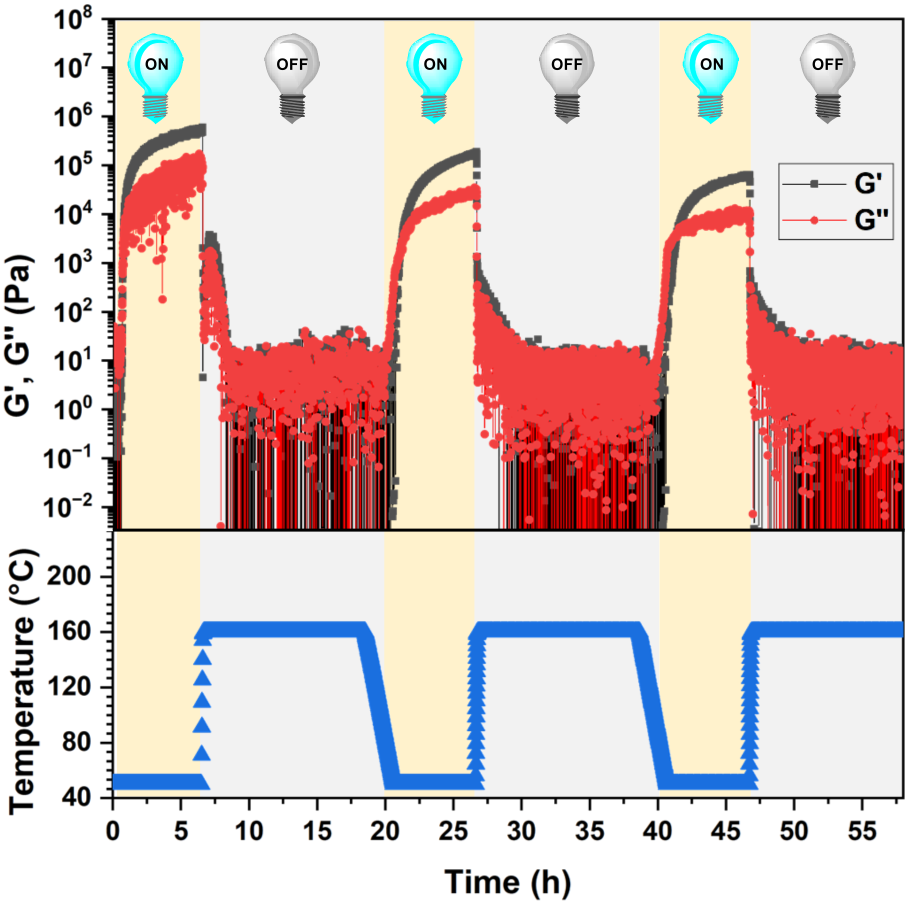

Figure S29. Thermal photorheology profile of **3-MTM** in bulk during consecutive cycles of UV irradiation (λ = 320 – 390 nm, 6 h) and prolonged heating (160 °C, 12 h). Irradiation cycles were performed at slightly elevated temperature (i.e. 50 °C) to aid sample preparation of the viscous monomer.

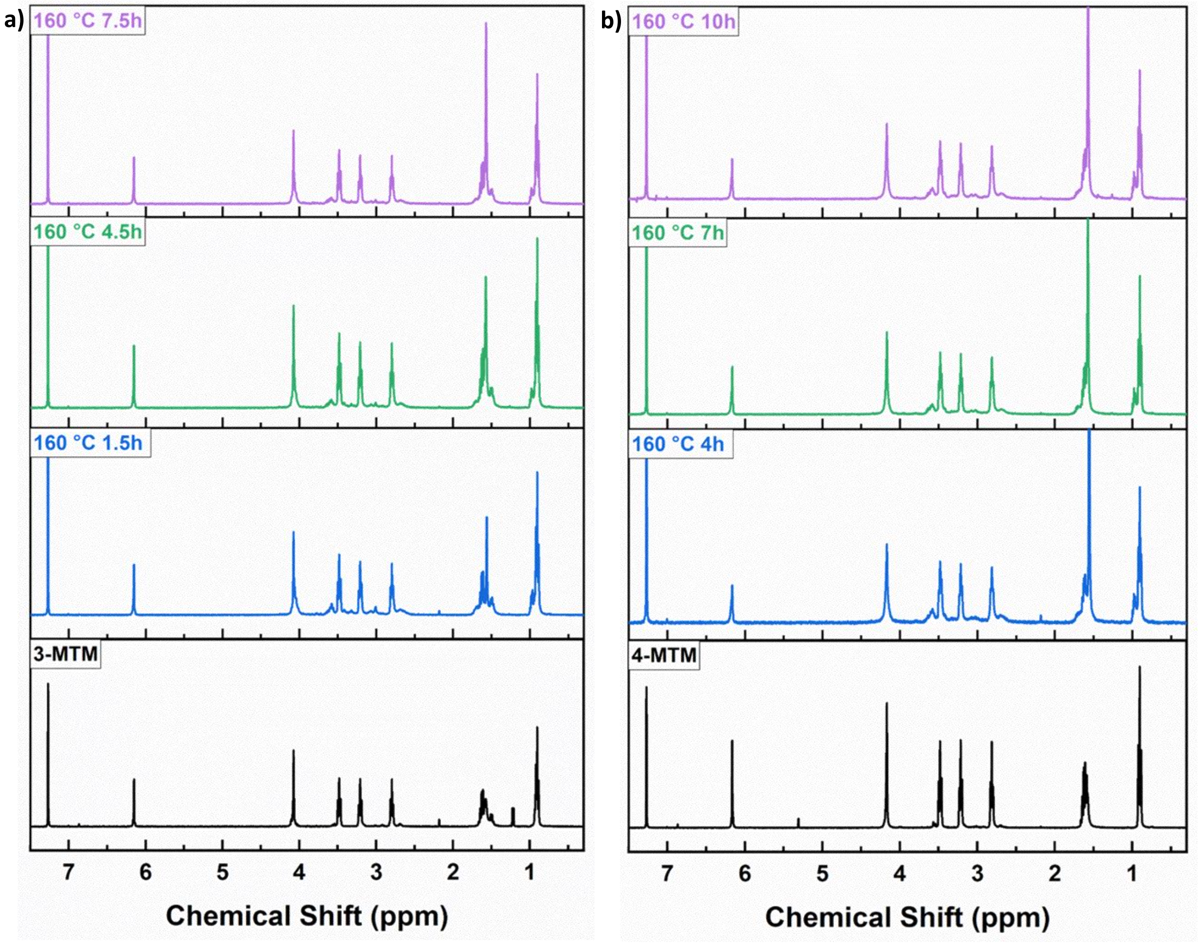

Figure S30. ^{1}H NMR spectra ($CDCl_3$) of (a) **3-MTM** and (b) **4-MTM** recorded during bulk decrosslinking of the corresponding networks at 160 °C, indicated a decreased presence of thiomaleimide substrates when heated beyond 4 hours.

Supplementary Methods

Instrumentation

Irradiation setups. Irradiation at λ = 365 nm was carried out using a Lumidox® II 96-well LED array. Spectral details are available on the supplier website (https://www.analytical-sales.com/product-category/lumidox-ii-led-arrays-lamps/lumidox-ii-led-arrays/). Samples were exposed to 15 mW cm^{-2} – 0.5 W cm^{-2} irradiance, measured using an S142C Integrating Sphere Power Sensor Head with Silicon Detector connected to a ThorLabs PM400 optical power meter calibrated at a wavelength of λ = 365 nm. Experiments conducted at λ_{max} = 254 nm were performed in a UVP Crosslinker instrument from Analytik Jena at an output setting of 500 mJ cm^{-2}.

Differential scanning calorimetry (DSC)/Thermogravimetric Analysis (TGA). Differential Scanning Calorimetry (DSC) and Thermogravimetric Analysis (TGA) measurements were carried out simultaneously using an SDT 650 from TA Instruments, equipped with a liquid nitrogen cooling system and an automatic sample robot. All measurements were carried out under a nitrogen atmosphere in standard 90 µL aluminium crucibles containing ca. 5 mg of compound material. Samples were heated from 25 °C to 800 °C at a rate of 10 °C min^{-1}. Thermally reversable networks were heated up to 100 °C to avoid decrosslinking during measurement. The resulting thermograms were analysed with the TRIOS software.

Differential scanning calorimetry (DSC). Differential Scanning Calorimetry (DSC) measurements were carried out on a Mettler-Toledo instrument, equipped with a liquid nitrogen cooling system and an auto-matic sampling robot. All measurements were carried out under a nitrogen atmosphere in standard 40 µL aluminium crucibles. DSC thermograms of pure MTM samples were measured from -110 to 200 °C at a heating rate of 5 °C min^{-1} for two heating-cooling cycles. DSC thermograms of fully cured MTM samples were measured from -100 to 100 °C at a heating rate of 5 °C min^{-1} for two heating-cooling cycles. The midpoint values were recorded as the glass transition temperatures (T_g).

an SDT 650 from TA Instruments, equipped with a liquid nitrogen cooling system and an automatic sample robot. All measurements were carried out under a nitrogen atmosphere in standard 90 µL aluminium crucibles containing ca. 5 mg of compound material. Samples were heated from 25 °C to 800 °C at a rate of 10 °C min^{-1}. Thermally reversable networks were heated up to 100 °C to avoid decrosslinking during measurement. The resulting thermograms were analysed with the TRIOS software.

Electrospray ionisation mass spectrometry (ESI-MS). Mass spectrometry was carried out on an Agilent 6130B ESI-Quad, fitted with autosampler and isocratic pump from an Agilent 1100. Samples were prepared in 80:20 methanol:water mixture. High resolution ESI-MS (HR-ESI-MS) spectra were recorded in positive mode using a Bruker Compact QToF MS with electrospray ionization (ESI). Samples were prepared in acetonitrile.

Infrared spectroscopy. Infrared spectra were recorded on a Bruker Alpha FT-IR spectrometer.

Nuclear magnetic resonance (NMR) spectroscopy. NMR spectra were recorded on a Bruker AV-300, HD-300, or HD-400 FT-NMR spectrometer. Measurements were conducted at 25 °C in the solvent as indicated. NMR results were analysed using the ACD/NMR Processor software. Chemical shifts (δ) are expressed in parts per million (ppm) with the residual solvent peak serving as an internal standard. Resonance multiplicities are abbreviated as follows: s (singlet), d (doublet), t (triplet), q (quadruplet), quint (quintuplet), sext (sextuplet) or m (multiplet).

Rheology. Rheology experiments were performed using an Anton Paar Modular Compact Rheometer (MCR) 302 rheometer equipped with a peltier P-PTD 200 bottom glass plate and an H-ETD 400 electrically heated temperature hood with compressed air cooling. Measurements were performed using a parallel plate configuration (PP25, 25 mm diameter) under a viscoelastic moving profile in oscillatory mode with a pre-set shear gap (typically between 0.2 mm and 0.1 mm), 0.1 % shear strain and 0.1 Hz angular frequency, whilst keeping the normal force constant (e.g. set at 0 N). Photocuring experiments were done using an OmniCure S2000 containing a high pressure 200 W mercury vapor short arc broad emitting UV-lamp equipped with either a 320 – 390 nm bandpass filter (estimated irradiance at λ = 365 nm of 62 mW cm^{-2}), or a 365 nm filter (7 W cm^{-2}). The incident light is guided through an optical fibre to the bottom of the glass plate thereby irradiation the sample from the bottom. Measurements were initially started at 50 °C to enable sufficient flow of the bulk material, needed for homogeneous sample loading. Following irradiation, the peltier plate and hood were heated to 160 °C over a time interval of 10 minutes. The resulting data was analysed using the RheoCompass™ software.

Thermogravimetric Analysis (TGA). TGA measurements were carried out using a Mettler-Toledo instrument, equipped with a liquid nitrogen cooling system and an automatic sample robot. All measurements were carried out under a nitrogen atmosphere in standard 40 µL aluminum crucibles. Samples were heated from 25 to 600 °C at a rate of 10 °C min^{-1}. The degradation temperature was derived as the temperature of 5% weight loss.

UV/vis spectrometry. UV/vis spectra were recorded at ambient temperature on an Agilent Technologies Cary 60 UV/vis. Absorbance was screened in the 200-800 nm wavelength range, using a standard cuvette with optical path length of 10 mm.

Materials

Bromomaleic anhydride (97 %, Fisher scientific), 1-propylamine (99 %, Aldrich), hexane-1-thiol (Aldrich), acetic acid (Merck), deuterated chloroform-*d* ($CDCl_3$, Aldrich), deuterated dimethylsulfoxide-d_6 (DMSO-d6, Aldrich), sodium acetate, anhydrous (Fisher scientific), *N*-ethylmaleimide (Aldrich), trimethylolpropane tris(3-mercaptopropionate) (Aldrich), pentaerythritol tetrakis(3-mercaptopropionate) (Aldrich).

All solvents and reagents were used as received from their supplier unless otherwise stated in the synthetic or experimental procedure.

Experimental procedures

Preliminary photo- and thermal reversion studies of thiomaleimide photodimer 1-MTM$_2$

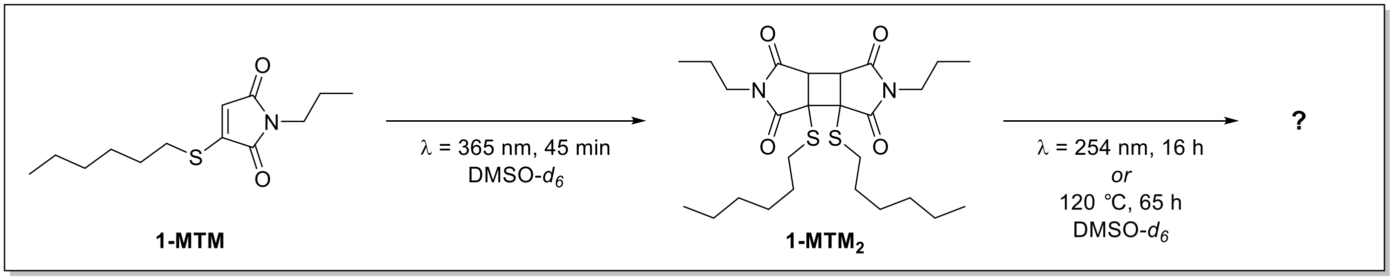

A solution of **1-MTM** (20 mg, 7.8 10^{-5} mol, 1.0 eq.) in 1.0 mL DMSO-*d*$_6$ was transferred into two NMR tubes (5 mm thin-walled borosilicate glass). The light-yellow solutions were irradiated for 45 min at λ = 365 nm with a UV LED array (15 mW cm^{-2}, Lumidox® II LED array). Offline ^{1}H NMR analysis confirmed complete consumption of **1-MTM** and the quantitative formation of the resulting photodimer **1-MTM$_2$**. A first tube containing photodimer **1-MTM$_2$** (10 mg in 0.5 mL DMSO-*d*$_6$) was irradiated for 16 hours at λ = 254 nm (500 mJ cm^{-2}, Analytik Jena UVP Crosslinker) and subjected to ^{1}H NMR spectroscopy to investigate the reversion into the initial **1-MTM** substrate. A second tube was placed in a pre-heated oil bath at 120 °C for 65 hours and subjected to ^{1}H NMR analysis, indicating a clean and complete reversion of **1-MTM$_2$** into **1-MTM**. The solution was subsequently re-irradiated for 60 min at λ = 365 nm with a UV LED array (15 mW cm^{-2}, Lumidox® II LED array), resulting in the reformation of **1-MTM$_2$** as evidenced from the corresponding ^{1}H NMR spectrum.

^{1}H-NMR (400 MHz, DMSO-*d*$_6$): δ (ppm) = 0.87 (m, 12H, C*H*$_3$), 1.24 (m, 12H, CH$_3$-(C*H*$_2$)$_3$), 1.41 (quin, *J* = 7.0 Hz, 4H, S-CH$_2$-C*H*$_2$), 1.58 (sext, J = 7.2 Hz, 4H, N-CH$_2$-C*H*$_2$), 2.67 (dt, *J* = 11.2, 7.3 Hz, 2H, S-C*H*$_2$), 2.93 (dt, *J* = 11.2, 7.3 Hz, 2H, S-C*H*$_2$), 3.47 (t, J = 6.9 Hz, 4H, N-C*H*$_2$), 3.51 (s, 2H, C*H*).

N-Ethylmaleimide reference experiments

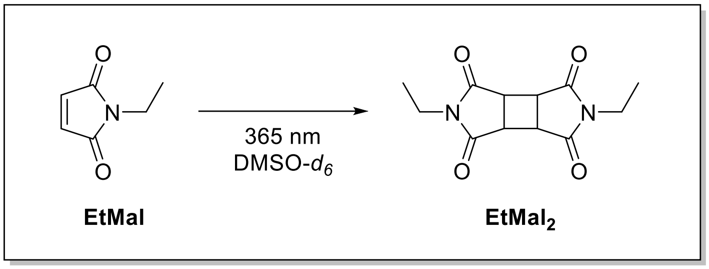

A solution of **EtMal** (3.12 mg, 2.5 10^{-5} mol, 1.0 eq.) in 0.5 mL DMSO-*d*$_6$ (50 mM) was transferred into an NMR tube. The tube was irradiated at λ = 365 nm with a UV LED array (0.5 mW cm^{-2}, Lumidox® II LED array) for up to 210 minutes. Periodic offline ^{1}H NMR measurements (DMSO-*d*$_6$) were conducted to monitor the progress of the photocycloaddition through the disappearance of the C=C*H* resonance at 7.01 ppm.

Photodimerization and cycloreversion kinetics

Aliquots of a 50 mM stock solution of **1-MTM** (127.6 mg, 5.0 10^{-4} mol, 1.0 eq.) in 10 mL DMSO-*d*$_6$ were distributed over several NMR tubes and irradiated for distinct periods of time with a UV LED array (λ = 365 nm, 0.5 W cm^{-2}). Photodimerisation kinetics were monitored via offline ^{1}H NMR measurements.

Next, 3 samples that showed complete conversion of **1-MTM** starting compound were used to obtain thermal reversibility profiles of **1-MTM$_2$** at three distinct temperatures, i.e. 140, 150 and 160 °C. For this, the resulting NMR tubes were placed in a pre-heated oil bath for distinct periods of

time. Once heated, the samples were cooled under running tap water and subjected to ^{1}H NMR analysis.

Determination of the cycloreversion rate coefficients and activation energy

The thermal reversibility profiles of the thiomaleimide photodimer **1-MTM₂** (main manuscript Fig. 3) express the fraction of remaining **1-MTM₂** upon heating at a well-defined temperature over time, i.e. 120 °C, 140 °C and 160 °C.

$$-\frac{d[M\boldsymbol{TM}_2]}{dt} = k[\boldsymbol{MTM}_2]$$

$$=> \ \ln[M\boldsymbol{TM}_2]_\mathrm{t} - \ln[M\boldsymbol{TM}_2]_0 = -\,kt$$

$$=> \ \ln\frac{[\boldsymbol{MTM}_2]_\mathrm{t}}{[\boldsymbol{MTM}_2]_0} = -\,kt$$

With the fraction of $\frac{[\boldsymbol{MTM}_2]_t}{[\boldsymbol{MTM}_2]_0}$ determined via integration of the ^{1}H NMR spectra as a function of time, the cycloreversion is observed to proceed with a first-order reaction rate coefficient *k*, which was determined at each investigated temperature using the least squares method linear interpolation.

Cycloreversion half-life times $t_{1/2}$ were calculated using:

$$t_{1/2} = \frac{\ln 2}{k}$$

Having established the relationship of *k* as a function of the temperature from the above observed first-order reaction kinetics, the activation energy E_a for the thermal reversion of thiomaleimide photodimer **1-MTM₂** was next determined from the Arrhenius equation.

$$k = Ae^{-\frac{E_a}{RT}}$$

$$=> \ \ln k = \frac{-E_a}{RT} + \ln A$$

By plotting ln k as a function of T^{-1}, an Arrhenius plot was obtained, from which the activation energy E_a was calculated using the least squares method linear interpolation (cf. Fig. S10).

$$y = ax + b$$

$$=> \ a = \frac{-E_a}{R}$$

$$=> \ E_a = -aR$$

With the gas constant R = 8.314 J K^{-1} mol^{-1} this gives E_a in J mol^{-1}. The error on the activation energy was determined by the uncertainty on the slope using the LINEST function in MS Excel.

Cyclability of MTM photodimerisation and thermal cycloreversion

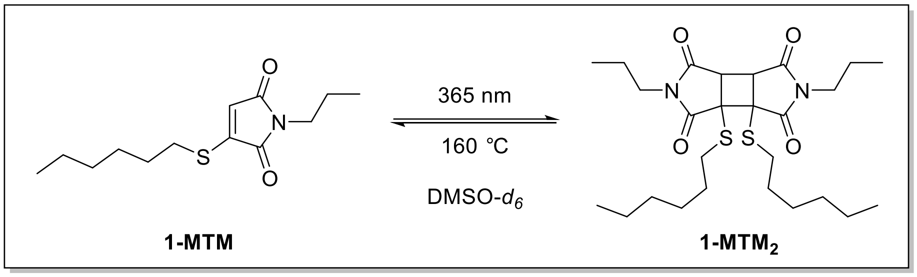

An NMR tube containing **1-MTM** (6.38 mg, 2.5 10^{-5} mol, 1.0 eq.) in 0.5 mL DMSO-d_6 (*cycle 0*) was irradiated for 30 min with a UV LED array (λ = 365 nm, 0.5 W cm^{-2}), cf. instrumentation section for specifications of the irradiation source. Following the period of irradiation (i.e., *cycle* $0^{1/2}$), the sample was transferred into a pre-heated oil bath at 160 °C and heated for 1.5 h. Irradiation and heating was continued for 5 consecutive cycles, with the extent of the photodimerisation and cycloreversion monitored after each step via offline ^{1}H NMR spectroscopy.

Reversible crosslinking of thiomaleimide materials

Crosslinking/de-crosslinking in solution for ^{1}H-NMR analysis

Diluted solutions of **3-MTM** and **4-MTM** (8 mg mL^{-1}, DMSO-d_6) were exposed to UV light (λ = 365 nm, 15 mW cm^{-2}). Structural changes were observed through offline ^{1}H NMR analysis, indicating full conversion of the multifunctional reagents into their corresponding [2+2] photo-cycloaddition products with characteristic ^{1}H NMR line broadening (Fig. 4 and Fig. S18). Further, UV/vis spectra of **3-MTM** and **4-MTM** confirmed thiomaleimide consumption upon irradiation by the observed disappearance of the $\pi \rightarrow \pi^*$ absorption band at λ_{max} = 354 nm (Fig. S19). Heating the resulting **3-MTM$_2$** and **4-MTM$_2$** photodimer solutions for 2 hours at 160 °C regenerated the thiomaleimide starting materials (Fig. 4 and Fig. S18, respectively).

Crosslinking/de-crosslinking in solution for gel formation

A solution of **3-MTM** (200 mg) in 1 mL DMSO in a glass vial was irradiated with UV LEDs at λ = 365 nm (30 mW cm^{-2}). Within 2 hours, photo-gelation was observed by means of a vial inversion test. The resulting crosslinked gel was placed in a pre-heated oil bath at 160 °C for 1.5 hours, resulting in the reformation of an orange-brown liquid. Reversible gelation of the resulting solution was demonstrated for a second cycle following re-irradiation (2 h, λ = 365 nm, 30 mW cm^{-2}) and subsequent heating (1.5 h, 160 °C).

Crosslinking/de-crosslinking in bulk for rheology measurement

The viscous **3-MTM** compound was loaded onto a 50 °C pre-heated glass peltier bottom plate of an Anton Paar MCR302 rheometer (cf. instrumentation section). The upper moving profile (parallel plate geometry) was lowered, the excess amount of the sample was trimmed and analysed in oscillatory mode in a closed upper heating hood thus preventing exposure to UV light during measurements. The rheology profile showing the evolution of G' and G" over time is depicted in Figure 5.

Synthetic procedures

Synthesis of thiomaleimide compounds

Synthesis of 3-(hexylthio)-1-propyl-1*H*-pyrrole-2,5-dione (3-(hexylthio)-*N*-propylmaleimide, 1-MTM)[1]

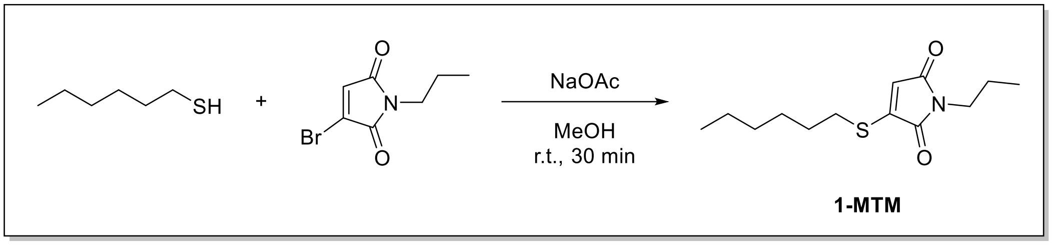

To a solution of *N*-propyl-3-bromomaleimide (3.0 g, 14.0 mmol. 1.0 eq. – synthesised according to a literature procedure[1]) in methanol (50 mL) was added sodium acetate (1.1 g, 14.0 mmol, 1.0 eq.). The solution was cooled to -78 °C. Then, hexane-1-thiol (2.3 ml, 14.0 mmol, 1.0 eq.) was slowly added dropwise under continuous stirring. After 10 minutes, the reaction mixture was evaporated to dryness *in vacuo* and the crude was purified by column chromatography (silica, eluent = petroleum ether:ethyl acetate 95:5). The title compound 3-(hexylthio)-*N*-propylmaleimide (**1-MTM**) was obtained as a pale yellow solid (2.64 g – 60 %).

^{1}H-NMR (400 MHz, DMSO-*d*$_6$): δ (ppm) = 0.80 (t, *J* = 7.4 Hz, 3H, N-CH_2-CH_2-CH_3), 0.87 (m, 3H, S-$(CH_2)_5$-CH_3), 1.27 (m, 4H, S-(CH_2)-CH_2-CH_2), 1.39 (quin, *J* = 7.2 Hz, 2H, S-CH_2-CH_2-CH_2), 1.49 (sxt, *J* = 7.3 Hz, 2H, N-CH_2-CH_2), 1.65 (quin, *J* = 7.4 Hz, 2H, S-CH_2-CH_2), 3.01 (t, *J* = 7.3 Hz, 2H, S-CH_2), 3.34 (t, *J* = 7.0 Hz, 2H, N-CH_2), 6.54 (s, 1H, C=C*H*). **^{13}C-NMR (100 MHz, DMSO-*d*$_6$):** δ (ppm) = 11.03 (CH_3), 13.83 (CH_3), 21.30 (CH_2), 21.95 (CH_2), 27.34 (CH_2), 27.80 (CH_2), 30.65 (CH_2), 30.81 (CH_2), 39.08 (CH_2), 118.19 (CH), 150.17 (C), 167.88 (C), 169.59 (C). **ESI-MS (m/z):** 278.3 $[M+Na]^+$.

Synthesis of 3-MTM

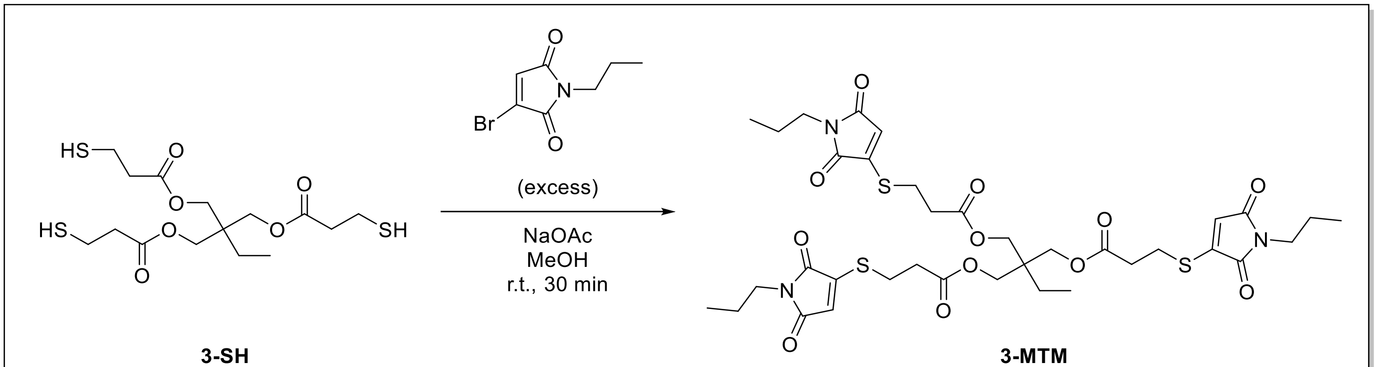

N-Propyl-3-bromomaleimide (7.00 g, 32.1 mmol, 4.5 eq. – synthesised according to a literature procedure[1]) and sodium acetate (2.63 g, 32.1 mmol, 4.5 eq.) were dissolved in 100 mL methanol. Then, trimethylolpropane tris(3-mercaptopropionate) (2.81 g, 7.13 mmol, 1.0 eq.) was added slowly. The reaction mixture was left to stir at ambient temperature for 30 minutes. Subsequently, the solvent was removed under reduced pressure and the crude product was dissolved in 100 mL dichloromethane and washed with water (3 x 100 mL). The organic phase was separated, and the solvent

removed *in vacuo*. The crude product was dissolved in a minimal amount of acetone and the obtained viscous product was precipitated in cold isopropanol to give a brown viscous product. The residue was dried in a vacuum oven at 40 °C overnight to obtain **3-MTM** as a brown-yellow viscous oil (4.0 g – 70 %).

^{1}H-NMR (400 MHz, DMSO-d_6): δ (ppm) = 0.75 - 0.86 (m, 12H, 4 x CH_3), 1.37 - 1.56 (m, 8H, 4 x CH_3-CH_2), 2.79 (t, J = 6.7 Hz, 6H, 3 x C(O)-CH_2), 3.22 (t, J = 6.8 Hz, 6H, 3 x S-CH_2), 3.34 (m, 6H, 3 x N-CH_2), 4.02 (s, 6H, 3 x O-CH_2), 6.62 (s, 3H, 3 x C=CH). **^{13}C-NMR (100 MHz, $CDCl_3$):** δ (ppm) = 7.28 (CH_3), 11.15 (CH_3), 21.80 (CH_2), 22.85 (CH_2), 26.16 (CH_2), 32.49 (CH_2), 39.82 (CH_2), 40.97 (C), 63.75 (CH_2), 117.90 (CH), 149.78 (C), 167.79 (C), 169.43 (C), 170.33 (C). **HR-ESI-MS (m/z):** *calc.*: 832.2214, *found*: 832.2227 $[M+Na]^+$.

Synthesis of 4-MTM

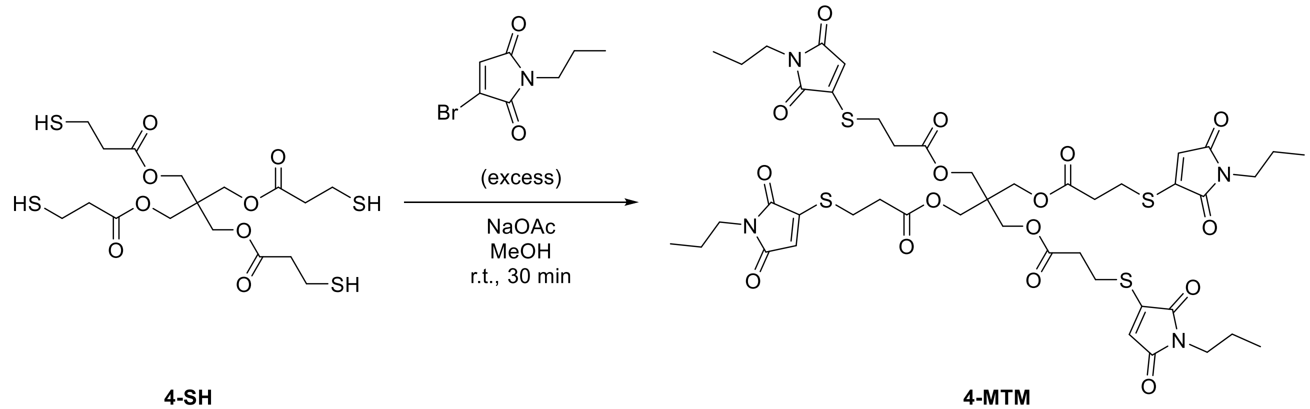

4-SH

4-MTM

N-Propyl-3-bromomaleimide (7.00 g, 32.1 mmol, 6.0 eq. – synthesised according to a literature procedure[1]) and sodium acetate (2.63 g, 32.1 mmol, 6.0 eq.) were dissolved in 100 mL methanol. Then, pentaerythritol tetrakis(3-mercaptopropionate) (2.61 g, 5.35 mmol, 1.0 eq.) was added slowly. The reaction mixture was left to stir at ambient temperature for 30 minutes. Subsequently, the solvent was removed under reduced pressure and the crude product was dissolved in 100 mL dichloromethane and washed with water (3 x 100 mL). The organic phase was separated, and the solvent removed *in vacuo*. The crude product was dissolved in a minimal amount of acetone and the obtained viscous product was precipitated in cold isopropanol to give a brown viscous product. The residue was dried in a vacuum oven at 40 °C overnight to obtain **4-MTM** as a brown-yellow viscous oil (3.6 g – 65 %).

^{1}H-NMR (400 MHz, DMSO-d_6): δ (ppm) = 0.80 (t, J = 7.4 Hz, 12H, 4 x CH_3), 1.49 (sxt, J = 7.3 Hz, 8H, 4 x CH_3-CH_2), 2.80 (t, J = 6.6 Hz, 8H, 4 x C(O)-CH_2), 3.22 (t, J = 6.8 Hz, 8H, 4 x S-CH_2), 3.34 (m, 8H, 4 x N-CH_2), 4.07 + 4.14 (2 x s, 8H, 4 x O-CH_2), 6.61 (m, 4H, 4 x C=CH). **^{13}C-NMR (100 MHz, $CDCl_3$):** δ (ppm) = 11.16 (CH_3), 21.80 (CH_2), 26.07 (CH_2), 32.41 (CH_2), 39.84 (CH_2), 42.43 (C), 61.83 (CH_2), 118.00 (CH), 149.62 (C), 167.80 (C), 169.42 (C), 170.15 (C). **HR-ESI-MS (m/z):** *calc.*: 1059.2466, *found*: 1059.2462 $[M+Na]^+$.

Supplementary references

(1) Tedaldi, L. M.; Aliev, A. E.; Baker, J. R. [2 + 2] Photocycloadditions of thiomaleimides. Chemical Communications **2012**, 48 (39), 4725-4727.
